# Pathological hemodynamic changes and leukocyte transmigration disrupt the blood–spinal cord barrier after spinal cord injury

**DOI:** 10.1186/s12974-023-02787-w

**Published:** 2023-05-20

**Authors:** Rubing Zhou, Junzhao Li, Zhengyang Chen, Ruideng Wang, Yin Shen, Rong Zhang, Fang Zhou, Yong Zhang

**Affiliations:** 1 Department of Orthopedics, Peking University Third Hospital, Beijing, 100191 People’s Republic of China; 2 Department of Neurobiology, School of Basic Medical Sciences and Neuroscience Research Institute, Peking University, Beijing, 100191 People’s Republic of China; 3 Key Laboratory for Neuroscience, Ministry of Education of China and National Health Commission of P.R. China, Beijing, 100191 People’s Republic of China; 4 PKU-IDG/McGovern Institute for Brain Research, Beijing, 100871 People’s Republic of China; 5 Eye Center, Renmin Hospital of Wuhan University, Hubei Wuhan, 430060 People’s Republic of China

**Keywords:** Spinal cord injury, Blood–spinal cord barrier, Two–photon microscopy, Tight junction, Hypothermia

## Abstract

**Background:**

Blood–spinal cord barrier (BSCB) disruption is a key event after spinal cord injury (SCI), which permits unfavorable blood-derived substances to enter the neural tissue and exacerbates secondary injury. However, limited mechanical impact is usually followed by a large-scale BSCB disruption in SCI. How the BSCB disruption is propagated along the spinal cord in the acute period of SCI remains unclear. Thus, strategies for appropriate clinical treatment are lacking.

**Methods:**

A SCI contusion mouse model was established in wild-type and LysM-YFP transgenic mice. In vivo two-photon imaging and complementary studies, including immunostaining, capillary western blotting, and whole-tissue clearing, were performed to monitor BSCB disruption and verify relevant injury mechanisms. Clinically applied target temperature management (TTM) to reduce the core body temperature was tested for the efficacy of attenuating BSCB disruption.

**Results:**

Barrier leakage was detected in the contusion epicenter within several minutes and then gradually spread to more distant regions. Membrane expression of the main tight junction proteins remained unaltered at four hours post-injury. Many junctional gaps emerged in paracellular tight junctions at the small vessels from multiple spinal cord segments at 15 min post-injury. A previously unnoticed pathological hemodynamic change was observed in the venous system, which likely facilitated gap formation and barrier leakage by exerting abnormal physical force on the BSCB. Leukocytes were quickly initiated to transverse through the BSCB within 30 min post-SCI, actively facilitating gap formation and barrier leakage. Inducing leukocyte transmigration generated gap formation and barrier leakage. Furthermore, pharmacological alleviation of pathological hemodynamic changes or leukocyte transmigration reduced gap formation and barrier leakage. TTM had very little protective effects on the BSCB in the early period of SCI other than partially alleviating leukocyte infiltration.

**Conclusions:**

Our data show that BSCB disruption in the early period of SCI is a secondary change, which is indicated by widespread gap formation in tight junctions. Pathological hemodynamic changes and leukocyte transmigration contribute to gap formation, which could advance our understanding of BSCB disruption and provide new clues for potential treatment strategies. Ultimately, TTM is inadequate to protect the BSCB in early SCI.

**Supplementary Information:**

The online version contains supplementary material available at 10.1186/s12974-023-02787-w.

## Background

Spinal cord injury (SCI) leads to long-term impairment and severe disability, but no effective clinical treatment is currently available. Soon after the primary injury, secondary injury further exacerbates the pathological damage. The blood–spinal cord barrier (BSCB) maintains the immune isolation of the spinal cord, keeping immunogenic substances and the innate immune response out of the nervous system [1]. BSCB disruption typically occurs as a result of traumatic impact during SCI [2, 3]. When the BSCB is disrupted, the spinal cord is exposed to the peripheral immune system [4]. Invasion of inflammatory substances from the blood triggers downstream pathophysiological cascades and aggravates secondary injury [3, 5]. Previous studies have shown that the BSCB remains chronically leaky after SCI, allowing for the infiltration of leukocytes [3, 6] and resulting in sustained neurodegeneration [7]. Therefore, BSCB disruption plays a pivotal role in the process of SCI. Several proteins forming endothelial paracellular junctions to maintain the BSCB, including tight junctions (TJs) and adherens junctions, contribute to the physical barrier properties of the BSCB [7]. TJs, mainly consisting of claudins, occludins, zonula occludens (ZO), and tricellulins, are the major complexes that maintain barrier properties and determine paracellular permeability [7]. In theory, barrier leakage can arise when abnormal alterations occur in the expression, localization, or post-translational modification of TJ proteins during injury [8]. Previous studies have reported that the expression of major TJs decreased at 8–24 h post-SCI [9–11]. However, very few studies have investigated the changes in TJs in the acute period of SCI, especially when BSCB leakage occurs [5].

The conventional view is that physical impact force directly disrupts the BSCB following spinal cord trauma [5, 12], while cellular damage, hemorrhagic and ischemic changes cause chronic BSCB breakdown and infiltration of immune cells [13]. In SCI, physical damage usually only impacts a limited region. However, multiple studies have demonstrated that BSCB disruption occurs in multiple spinal segments that are indirectly damaged and can even spread along the entire length of the spinal cord [14, 15]. Here, we aimed to explore how primary physical injury leads to widespread BSCB disruption in the acute phase after SCI.

Nevertheless, no effective method has been developed specifically to protect the BSCB or reduce barrier leakage after injury [1]. The lack of intervention methods also hinders further exploration of the mechanism of BSCB disruption in SCI. Target temperature management (TTM) is used to intentionally cool the core body temperature below the normal level to slow down multiple pathological processes and has been clinically applied as an adjuvant treatment for SCI in many hospitals [16, 17]. Multiple studies have observed that managing the core temperature to 33 °C for 4 h could reduce barrier leakage and attenuate local inflammation in brain trauma [18–21]. This therapeutic mechanism is also expected to exist in the spinal cord after SCI [22, 23]. Some reports have indicated that TTM could decrease barrier permeability and tissue edema in human SCI patients [24]. However, the underlying mechanism has not been fully verified. In our study, we utilized TTM to prevent BSCB disruption and examined the efficacy of TTM on BSCB, with a similar procedure applied in brain trauma.

Previous studies on BSCB disruption were mainly based on postmortem studies [5, 25], causing some factors to be overlooked. To obtain a more comprehensive understanding of BSCB disruption, we utilized two-photon microscopy to image the injured spinal cord in vivo longitudinally. Our results demonstrate that the extensive BSCB disruption is mainly a secondary change after the primary physical damage of SCI. While no changes in the expression of the main TJ proteins were detected 4 h post-SCI, the widespread formation of gaps in TJs could be responsible for the impending BSCB leakage in acute SCI. Pathological hemodynamic changes and rapid infiltration of leukocytes induce extensive gaps in TJs and barrier leakage at small vessels. TTM treatment showed little protective effect on BSCB impairment in the acute period after SCI. Widespread BSCB disruption is a secondary change that suggests a potential therapeutic window for clinical intervention before extensive BSCB disruption, and secondary injury-related factors may be new targets to protect the BSCB in SCI. Our study could pave the way for a better understanding of the role of BSCB disruption in the acute period after SCI and provide potential strategies for SCI treatment.

## Methods

### Animals

Wild-type C57BL/6 mice were purchased and used at the Department of Laboratory Animal Science of Peking University Health Science Center. *LysM*-YFP mice were provided by Dr. Zhongjun Dong’s laboratory at Tsinghua University (Beijing, China). *Tie2*-tdTomato mice were provided by Dr. Lemin Zheng’s laboratory at Peking University Health Science Center (Beijing, China). Filial generation mice were genotyped before being included in the experiment. Experiments were conducted on adult male mice (23 ± 1 g) at 8–10 weeks. The mice were given ad libitum access to food and water and housed in cages on a 12-h day/night cycle. Wild-type and transgenic mice from the same cage were randomly assigned into the respective groups.

### Surgical preparation

Mice were anesthetized with ketamine/xylazine (100/10 mg/kg, i.p.). The anesthetic was changed to pentobarbital (70 mg/kg, i.p.) in measuring the hemodynamic parameters to minimize the influence of anesthesia. The mice were placed on a feedback-controlled heating plate connected to a rectal probe to maintain the core body temperature at the target level. The systemic temperature was maintained at normothermia (NT, 37.0 ± 0.5 °C) or target temperature management (TTM, 32.0 ± 0.5 °C) in all experiments. 32 °C of TTM was chosen by balancing the safety and efficacy in our previous data, which is similar to the optimized temperature reported by the University of Miami [26]. A single-level bilateral laminectomy was performed with Vanna microscissors to expose the spinal cord at the T12 level. Animals that suffered an accidental injury during the operation were excluded. The blood was wiped away, and a sterile medical gel foam soaked with prewarmed saline was placed on the spinal cord. Blood pressure was measured using a noninvasive blood pressure system (CODA Monitor, Kent Scientific) equipped with inflation–deflation tail cuffs. The blood pressure measurement session was averaged from 8 repeat measurements to represent each time point.

### Contusion SCI

Mice were immobilized by clamping the vertebral column to the platform, and the dura mater was not removed. Contusion injury was delivered to the exposed spinal cord with a commercial NYU standardized gravity impactor (RWD Life Science, 68097) equipped with a 6.5 g metal impactor with a 2.0 mm semiglobular tip that dropped through the guide rail from a height of 5.4 cm. SCI was induced in wild-type and transgenic mice in the NT and TTM groups, respectively. The control mice received laminectomy with an intact spinal cord.

### In vivo imaging

A customized image chamber was slightly modified from the apparatus described by Farrar et al. [27]. In brief, a top plate with a 5-mm hole for a coverslip was screwed to two metal bars placed on both sides of the spine. Kwik-Sil silicone elastomer (World Precision Instruments, WPI) was applied to the surface of the spinal cord to close the chamber under a coverslip. Kwik-Cast sealant (WPI) or dental cement was swabbed to seal the chamber on the skin. Resealing the coverslip usually takes more than 15 min after the mice receive SCI and before the subsequent imaging proceeds. Alternatively, another homemade imaging chamber was used to achieve rapid imaging. The animal was stabilized on the platform, and dental cement was applied around the wet gel foam to build a dam. The chamber was infused with prewarmed saline at the target temperature. After imaging the intact spinal cord, the animal with the fixing device was returned to the imaging platform after receiving SCI. Vascular topography was imaged with a CCD camera to relocate the position. The first images post-SCI were acquired between 2 and 4 min after SCI on this homemade imaging chamber.

### Pharmacological or fluorescent materials application

The nitric oxide donor diethylamine-NONOate (EMD Millipore) was used to induce vasodilation and slow blood flow. Fresh 1.0 μM NONOate solution was injected into the homemade chamber for in vivo imaging. Gelatin foam soaked with fresh 1.0 μM NONOate solution was applied to the injured spinal cord and changed every 15 min before the spinal cords were harvested 4 h post-SCI. Colibacillus lipopolysaccharide (LPS) was injected intraperitoneally at a 5 mg/kg dose 12 h before two-photon imaging. In another group, the spinal cords were harvested for immunofluorescence 24 h after injection of LPS to allow for model stabilization. The CXCR2 antagonist SB225002 (4 mg/kg, Merck) was injected intraperitoneally 2 h before the mice underwent SCI.

All fluorescent substances were injected intravenously 30 min before the first images were captured. The following reagents were used at the indicated concentrations in our study: 40 kDa TRITC-dextran, 150 kDa FITC-dextran (Sigma, 5% w/v in saline, 100 μL), and PE- or Alexa Fluor 700-conjugated rat anti-mouse GR-1 (Ly6G/C) antibody (BD Biosciences, 25 μg/kg). The antibody was washed twice with phosphate-buffered saline (PBS) using a 10 kDa centrifugal filter (Millipore) to remove sodium azide before injection.

### Two-photon microscopy

Two-photon imaging was performed with a Leica TCS SP8 DIVE microscope equipped with a Mai Tai DeepSee pulsed laser (Spectra-Physics). Imaging was performed under an HCX IR APO L25 × /0.95 water immersion objective (Leica). GFP or YFP was excited at 850 nm, FITC or tdTomato was excited at 960 nm, TRITC or PE was excited at 1050 nm, and Alexa Fluor 700 and TRITC were excited at 860 nm. Images of 512 × 512 pixel fields were acquired for data collection and of 1024 × 1024 pixel fields for use as representative images (area of 738 × 738 μm^2^). Identical imaging conditions were used for paired groups. Images used as landmarks and time-lapse movies were captured under a Leica DFC425 CCD camera.

### Calculation of hemodynamic parameters

Wild-type mice were intravenously injected with 150 kDa FITC-dextran via the tail vein or retro-orbital sinus 30 min before imaging. Parameters were measured and analyzed in LasX (Leica software). The mean diameter was measured from the maximum intensity projection of images with a z-step of 0.6 μm at the ROI. Vessels submerged in leaked fluorescence post-SCI were excluded from the analysis. The average blood flow velocity was measured by the x-t line-scan method. The ROI was set as 100 μm in length, and the venules in the ROI were regarded as cylindrical pipes to simplify the calculation. The volumetric flow was calculated by the Radon transform algorithm:

1 $$\mathrm{volumetric\,flow }= 1/2 \times \pi {\times \mathrm{radius}}^{2}\times {\mathrm{velocity}}_{\mathrm{max}}$$

The pressure drop was calculated by the Poiseuille formula:

2 $$\mathrm{pressure\,drop}=\mathrm{volumetric\,flow}\times \mathrm{flow resistance}=4\times \mathrm{viscosity}\times \mathrm{length}\times \frac{{\mathrm{velocity}}_{\mathrm{max}}}{{\mathrm{radius}}^{2}},$$

3 $$\mathrm{flow\,resistance}=8\times \mathrm{viscosity}\times \mathrm{length}/\left(\uppi \times {\mathrm{radius}}^{4}\right).$$

The shear rate and shear force were calculated by the Newton inner friction law:

4 $$\mathrm{shear\,rate}=8\times \frac{\mathrm{velocity}}{\mathrm{diameter}};$$

5 $$\mathrm{shear\,force}=\mathrm{viscosity}\times \mathrm{shear\,rate}.$$

The viscosity (η) was regarded as a constant here because blood viscosity is mainly determined by the hematocrit [28]. We arbitrarily set the viscosity to 3 × 10^–3^ Pa.s (physically 2.0 × 10^–3^–4.0 × 10^–3^ Pa.s) in the calculation. Shear force is expressed in dynes per centimeter square (dyn/cm^2^; 1 Pa = 10 dyn/cm^2^).

### Quantification of fluorescent density

40 kDa TRITC-dextran was intravenously injected into wild-type mice 30 min before imaging. 3D projections with a z-step of 5 μm were reconstructed in LasX (Leica). The extravascular integrated density was measured in ImageJ (NIH).

### Calculation of leukocyte number

The number of leukocytes was automatically counted in Imaris 9 (Bitplane) with a spot radius greater than 4 μm. The luminal volume of vessels was measured in 3D projections in LasX (Leica). In the confocal image, the number or area of leukocytes was determined by particle analysis in ImageJ (NIH). The same intensity threshold and spot radius were applied for each image.

### Laser-doppler flowmetry

Blood perfusion of the spinal cord was measured with the PeriCam PSI system (Perimed, Stockholm) with a noncontact laser speckle contrast CCD camera. The blood perfusion was averaged by the time session and recorded every 5 min. Prewarmed saline was manually dropped onto the spinal cord. Data were collected and analyzed in PIM-Soft (Perimed).

### Immunohistochemistry

Animals were deeply euthanized with pentobarbital and then transcardially perfused with 6 U/mL of heparin in PBS and 4% paraformaldehyde (PFA). The immediate time point for immunofluorescence staining meant mice were held 0 min post-SCI before being humanely sacrificed. The sample harvesting procedures took at least 8 min. T11-L1 spinal cord segments (wounded and adjacent spinal segments, the same below) were harvested, postfixed overnight in 10% formalin at 4 °C, and dehydrated in gradient sucrose solutions. The sectioned tissue slices were blocked with 2% PBST (2% Triton X-100 in PBS) and 5% goat serum for 30 min at 37 °C and then incubated in primary antibody diluted in 0.4% PBST (0.4% Triton X-100 in PBS) with 1.5% goat serum for two nights at 4 °C. The sections were washed and incubated with secondary antibodies and DAPI (Applygen) diluted in 0.4% PBST with 1.5% goat serum overnight at 4 °C. For immunofluorescence staining of leukocytes, the sections were blocked with 0.4% PBST with 10% goat serum for 2 h, incubated in primary antibody diluted in 0.4% PBST with 10% goat serum overnight at 4 °C, and incubated in secondary antibodies and DAPI for 2 h at room temperature. Negative control slides were incubated in 0.4% PBST containing 10% goat serum without primary antibody. A detailed description of the antibodies is presented in Additional file 1: Table S1.

Images were taken with a Leica TCS STED microscope equipped with a 40 × HC PL APO CS2 oil-immersion lens (NA 1.3). Images of TJs were captured in 512 × 512 pixel fields (spatial resolution of 138 × 138 μm^2^, 0.5 μm increment). Noncapillary vessels (diameter ranging from 10–30 μm) inside the tissue were chosen to avoid artificial lesions. The gaps were manually measured and counted with LasX software (Leica software) in a double-blind manner. Images of leukocytes were captured in 1024 × 1024 pixel fields (spatial resolution of 250 × 250 μm^2^, 1 μm increment) at the boundary of the white and gray matter.

For immunohistochemical staining of IgG, every tenth Sect. (8 μm thick) was harvested and incubated in 3% hydrogen peroxide with methanol for 30 min, 10% blocking serum for 2 h, and primary anti-IgG antibody (Bethyl) overnight at 4 °C. After washing, the sections were incubated with HRP-conjugated secondary antibody and detected with a 3’-diaminobenzidine (DAB) substrate kit (Zsbio). Negative control slides were incubated in 10% donkey serum without primary antibody. The sections were autoscanned with the NanoZoomer Digital Pathology system (Hamamatsu), and the images were analyzed in ImageJ (NIH).

### Whole-tissue clearing (uDISCO)

The protocol was slightly modified from a previously published version [29]. The spinal cords were harvested from *LysM*-YFP mice at 6 h post-SCI because the size and shape of the lesion core appeared inconsistent at 4 h. The tissues were postfixed in 10% neutral formalin for 24 h at 4 °C. The tissues were dehydrated in gradient tert-butanol and puretert-butanol (Sigma) for 12 h each at 34–35 °C. Then, the sections were incubated in benzyl alcohol:benzyl benzoate:diphenyl ether (BABB-D4, 4:8:3, Sigma) overnight.

The cleared samples were imaged the following morning with a Zeiss light-sheet Z.1 microscope (Zeiss) equipped with two 5 × /NA 0.16 imaging objectives and a 5 × /NA 0.1 illumination objective. YFP was excited by 100% intensity at 514 nm and received with a 575–615 nm filter. For imaging at 2.5 × magnification, a 702.33 × 702.33 μm^2^ (1920 × 1920 pixels) area of each tissue was scanned with a 3.25 μm step size. Image stacks acquired by continuous scanning without the contrast-blending algorithm were reconstructed with Zen 2014 SP1 (Zeiss software) and Imaris Voloom (Bitplane Imaris software). The spots were automatically counted in a 500 × 500 × 500 μm^3^ cube in Imaris 9 (Bitplane Imaris software) by setting the long axis of leukocytes to greater than 8 μm.

### Automatic capillary western blotting

TJ proteins and CD31 levels were measured with an automatic capillary western blot system (Simple Western, ProteinSimple) according to the manufacturer’s instructions. In brief, the membrane component was extracted with a Mem-PER Plus kit (Pierce) and diluted to the appropriate concentration with master mix (ProteinSimple). The samples were incubated at 95 °C for 5 min for denaturation. Oligomeric claudin-5 and occludin were denatured at 37 °C for 30 min to unfold the oligomeric structure to obtain a single band. Each sample and the reagents were sequentially loaded into a 12–230 kDa assay well plate, except ZO-1, which was loaded into a 60–440 kDa plate. Vinculin was used as a loading control for ZO-1 and ATPase for the others. The levels of reference proteins were much greater than the target proteins, so the detection of reference protein and target protein was not performed in one well of the plate. ZO-1 cannot be extracted from the membrane or plasma component, so ZO-1 expression was evaluated in whole lysed tissue. The molecular weight of the proteins on the liquid gel (the composition was not provided, but the manufacturer stated that it did not contain SDS) was slightly different from traditional SDS gels [30]. The molecular weights of all proteins except for tricellulin were verified with additional primary antibodies. The manufacturer confirmed the molecular weights according to the manufacturer’s antibody vocabulary. The peak chemiluminescence intensity was computed by Compass software (ProteinSimple). Virtual images of the lanes were used as representative images (Additional file 7). Information for all antibodies is presented in Additional file 1: Table S1.

### Statistics

All statistical analyses were performed in SPSS (IBM software) or Prism 9 (GraphPad software). Data assessment and analysis were performed by researchers blinded to the experiments. No statistical methods were used to predetermine the sample sizes. Assumptions (normality, equal variance) were tested before performing parametric analyses. Multiple group comparisons were conducted by one-way ANOVA followed by a post hoc Bonferroni analysis or the Kruskal–Wallis test for nonparametric data. Elapsed time data were analyzed by two-way ANOVA followed by a post hoc Bonferroni analysis or general estimated equation for nonparametric data. No data were excluded from the statistical analysis. Details of the statistical results for specific experiments, including the exact n numbers, precision measures, statistical tests, and definitions of significance, are presented in the figure legends. Generally, the data are expressed as the mean ± SEM; n.s., nonsignificant, *P < 0.05, **P < 0.01, ***P < 0.001, ****P < 0.0001 in all figures. The detailed statistical results are provided in Additional file 6: Table S2.

## Results

### Diffused leakage of blood-derived substances indicates widespread BSCB disruption post-SCI

**Fig. 1:**
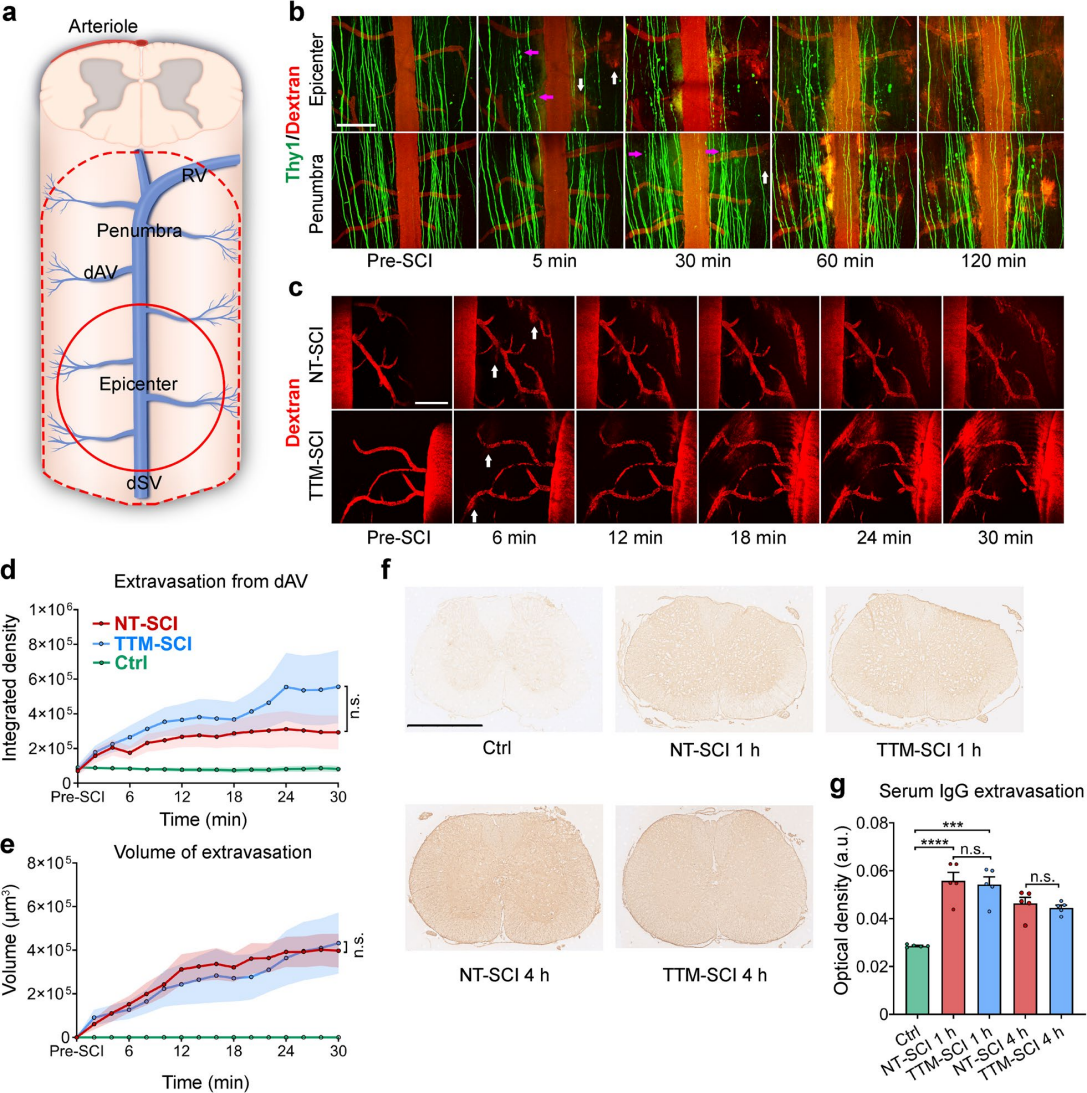
Extensive blood-derived component extravasation indicates widespread BSCB disruption after SCI. **a** Schematic drawing of vessels in the murine spinal cord. The primary physical impact (epicenter, red line) typically causes more extensive secondary injury (penumbra, red dashed line). *dAV* dorsal ascending venule, *dSV* dorsal spinal vein, *RV* radial vein. **b** Time-lapse imaging of *Thy1*-GFP mice after acute SCI. Leakage of 40 kDa TRITC-dextran (white arrow) was detected in the epicenter (upper panel) several minutes post-SCI. Leakage and axonal degeneration (red arrow) were detected in the penumbra region (lower panel) after approximately 30 min (*n* = 4 independent experiments). Scale bar = 200 μm. **c**–**e** Extravasation of dextran (white arrow) in the epicenter during normothermia (NT, upper panel) and TTM (lower panel). Scale bar = 200 μm. Quantification of the fluorescence intensity (**d**) and volume (**e**) of dextran extravasated from the dAV in the epicenter in (**c**) (*n* = 10 mice, or *n* = 6 in the ctrl group). **f** Representative immunohistochemical images of serum IgG extravasation in the penumbra area from adjacent nonimpacted spinal segments. Scale bar = 1 mm. **g** Optical density analysis of leaked IgG in multiple spinal segments at 1 and 4 h post-SCI (*n* = 5 mice). Data are presented as the mean ± SEM; ***P < 0.001, ****P < 0.0001; nested, general estimated equation (**d**, **e**), one-way ANOVA (**g**)

BSCB disruption leads to extravasation of blood-derived immunogenic substances into the spinal cord, exacerbating secondary injury following SCI [31]. To monitor the dynamic process of BSCB disruption post-SCI, mice were intravenously injected with 40 kDa TRITC-dextran, and time-lapse imaging was performed using two-photon microscopy. After laminectomy, a homemade imaging installation was used to monitor the same location before and after SCI (Additional file 1: Fig. S1a). The contusion-SCI model was employed because this model is more clinically relevant and closely mimics SCI in patients [13, 32]. Contusion injury was induced on the T12 spinal cord with a New York University (NYU) weight-drop impactor [33]. The epicenter was defined as the area that was directly impacted, and the penumbra was defined as the area that was not physically impacted but experienced secondary injury (Fig. 1a). In mice, only the collecting venous vessels were located on the dorsal side of the spinal cord, where the physical impact occurred [34]. It is currently technically unfeasible to image the main arterial vessels on the ventral side, so we mainly focused on the venous vessels in vivo. The primary injury did not induce apparent hemorrhage or topographical changes in the blood vessels, while fluorescent substance leakage was detected in the epicenter within several minutes post-SCI (Fig. 1b, upper panel). The first images were acquired between 2 and 4 min (average 3 min) after the impactor hit the spinal cord (Additional file 1: Fig. S1b, c). To assess the injury process in detail, we simultaneously imaged the region approximately 2 mm away from the epicenter, which was considered the penumbra region. Within approximately half an hour, leakage occurred around vessels in the penumbra area, where axons also started to degenerate (Fig. 1b, lower panel), indicating that BSCB disruption had spread. Leakage of hematogenous substances was predominant around the dorsal spinal vein (dSV) and dorsal ascending venules (dAVs) (Fig. 1c), suggesting that venules were susceptible to injury [35, 36]. Thus, we mainly quantified extravasation through venules in the epicenter (Additional file 1: Fig. S1d). Imaging was performed for 30 min because the vascular boundary was indiscernible at later time points. Compared to normothermia (NT), TTM did not show any beneficial effects on the extravascular fluorescence density or leakage volume (Fig. 1d, e; Additional file 1: Fig. S1e).

In vivo imaging could not reveal changes in the whole tissue due to the limited imaging depth. Previous studies have also shown that fluorescent dextran can be engulfed by myeloid-derived cells or glia during imaging [37–39]. Thus, we performed immunohistochemistry on transverse sections to verify the degree of BSCB permeability by measuring the leakage of nonspecific immunoglobulin G (IgG). It is known that B lymphocytes are not present in the normal neural parenchyma and only start to infiltrate the parenchyma several hours post-SCI; thus, the immunoglobulins present in the parenchyma are considered to derive from the serum [40, 41]. Extensive leakage of IgG was observed in the injured and adjacent spinal segments at 1 and 4 h post-SCI (Fig. 1f, g). Leakage of IgG into the parenchyma did not further increase at 4 h compared to 1 h after SCI. Some cysts were observed in the gray matter near the epicenter at 4 h (Additional file 1: Fig. S1f). TTM treatment did not show any effects in preventing IgG leakage in multiple spinal segments. These results show that primary injury leads to rapid BSCB disruption in the epicenter within several minutes post-SCI and that the extent of BSCB disruption gradually increases over time.

### Widespread structural damage to paracellular junctions after SCI

**Fig. 2:**
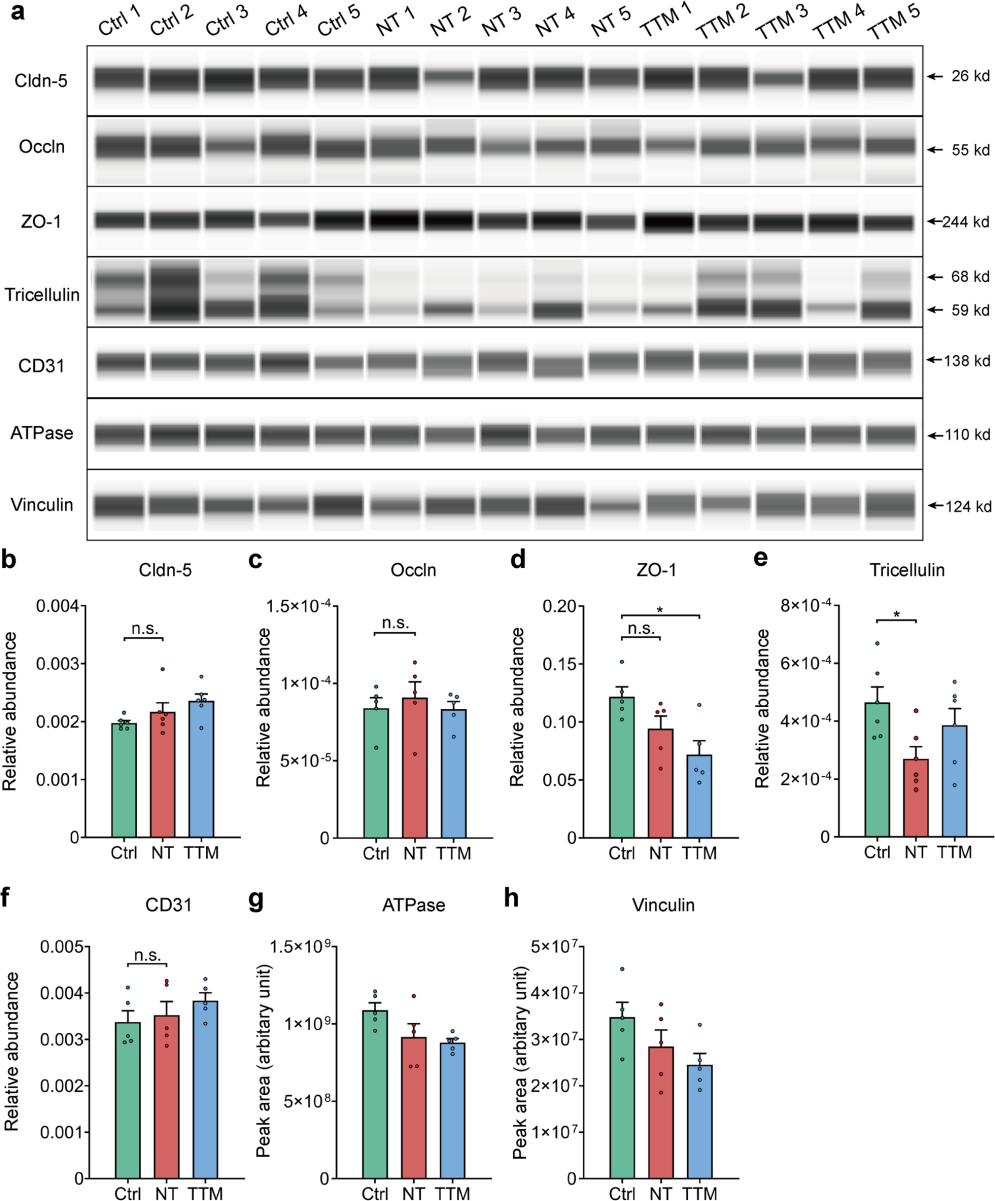
Membrane expression of major tight junction (TJ) proteins remains unaltered during BSCB leakage. **a** Capillary western lane view images of junctional proteins. **b**–**h** Quantification of the relative expression of the main TJ proteins 4 h post-SCI with NT and TTM. The concentration of each protein was calculated from the peak area in the data provided in Additional file 1: Fig. S2. (**b**) claudin-5 (cldn-5); (**c**) occludin (occln); (**d**) ZO-1; (**e**) tricellulin; (**f**) CD31; (**g**) ATPase; (**h**) vinculin. Vinculin was used as a loading control for ZO-1, and ATPase for the other proteins (*n* = 5 mice). Data are presented as the mean ± SEM; *P < 0.05; nested, one-way ANOVA (**b**-**h**)

The mechanism of BSCB disruption during the early period of SCI has not been fully investigated. It is generally believed that the majority of extravasation occurs in a paracellular manner, while the transcellular pathway accounts for only a small portion of extravasation [8, 42]. TJs, the major paracellular junctional components of the BSCB, seal the junctions between endothelial cells and determine paracellular permeability [1]. However, limited studies have focused on TJs in the early period after SCI, when BSCB disruption occurs. The widespread barrier leakage may hint at a functional or structural breakdown of paracellular junctions at the BSCB. To determine whether junctional proteins deteriorate post-SCI, we next examined the expression of TJ proteins, including claudin-5, occludin, ZO-1, tricellulin, and an adherens junction protein CD31 (PECAM-1). Traditional immunoblotting requires isolating the microvessels from the samples pooled together for not being sensitive enough to adequately measure the levels of TJ proteins [43, 44]. We instead utilized a capillary western blot system (Simple Western™) for immunoblotting analysis (Additional file 1: Figs. S1g and S2). Only tricellulin, which is only located at the junctions of three endothelial cells as a substitute for occludin [45, 46], showed a decrease in membrane expression 4 h post-SCI (Fig. 2), and this reduction in tricellulin expression was reversed by TTM (Fig. 2e). Interestingly, a decrease in the expression of ZO-1 was detected during TTM after SCI (Fig. 2d). The data showed no major change in the expression of TJ proteins 4 h post-SCI except for tricellulin, while TTM had mixed effects.

**Fig. 3:**
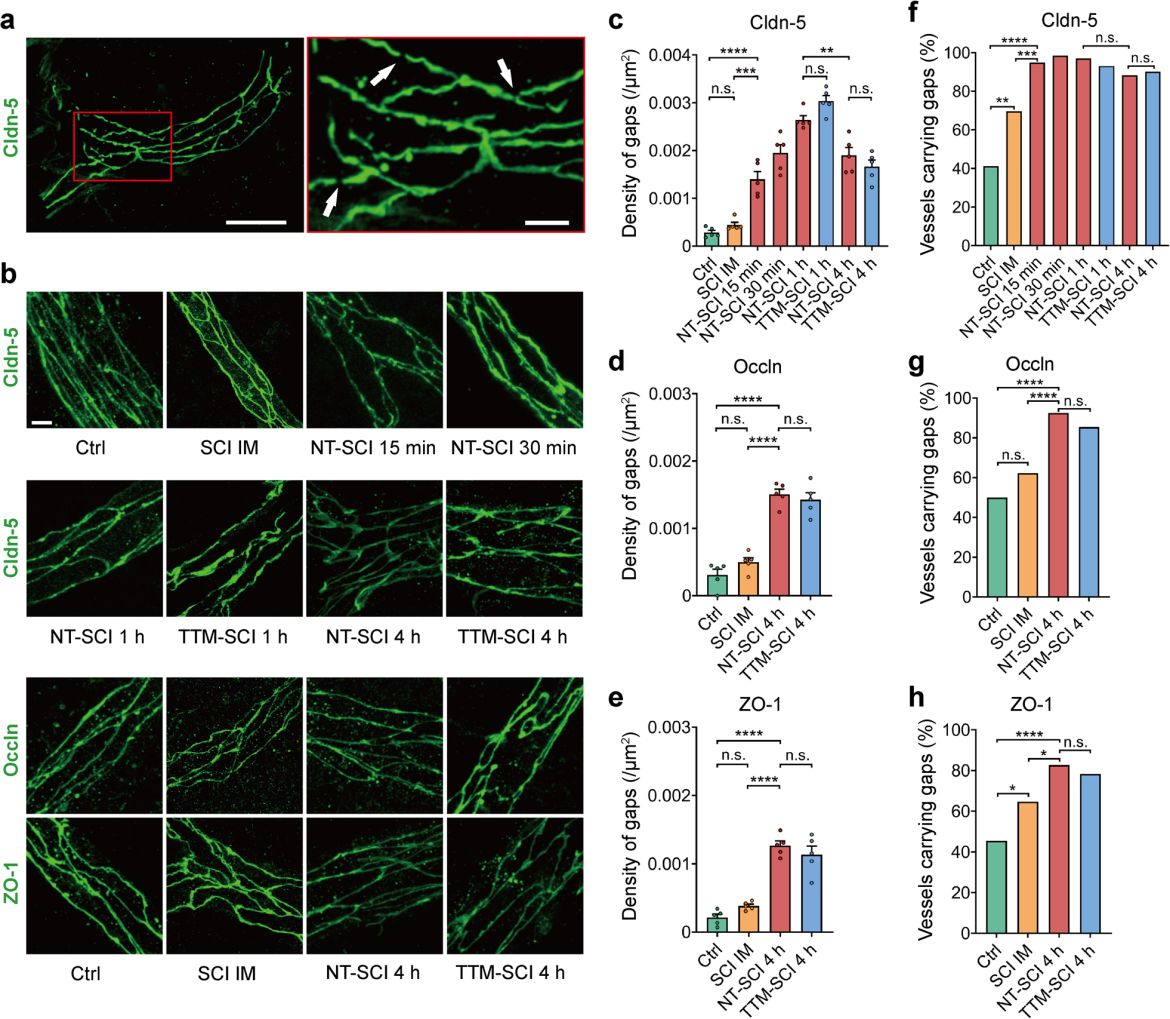
Multiple gaps form in TJs at noncapillary small vessels post-SCI. **a** Gap formation (arrows) in TJs at small vessels post-SCI. Scale bar: left = 20 μm, right = 5 μm. **b** Magnification of representative immunofluorescence images of claudin-5, occludin and ZO-1. IM, immediate. The immediate time point for immunofluorescence staining meant mice were held 0 min post-SCI before being sacrificed. **c** Quantification of the gap density at claudin-5 at different time points with NT or TTM post-SCI. **d**, **e** Quantification of the gap density at occludin (**d**) and ZO-1 (**e**) at vessels 4 h post-SCI with NT or TTM. **f**–**h** The proportion of vessels showing gaps in claudin-5 (**f**), occludin (**g**) and ZO-1 (**h**) among the total vessels in **c**-**e**. *n* = 5 mice; a total of 51, 69, 58, 63, 68, 44, 51, 51 vessels in **c**; a total of 97, 129, 105, 115 vessels in (**d**, **e**). Data are presented as the mean ± SEM; *P < 0.05, **P < 0.01, ***P < 0.001, ****P < 0.0001; nested, one-way ANOVA (**c**–**e**); chi-square test (**f**–**h**)

The expression of major TJ proteins remained unchanged at 4 h post-SCI, which could not account for the rapid and widespread leakage. Next, we performed immunostaining for TJ proteins accompanied by CD31, which is also a common marker of vessels [47], to assess the structural changes in paracellular junctions following SCI (Additional file 1: Fig. S3). Physically, TJs are weaker on venules, where plasma proteins and leukocytes prefer to extravasate during inflammation [48, 49], which agrees with our findings that leakage was predominant around venules after SCI (Fig. 1c). We aimed to focus on small noncapillary venules, but it would be inappropriate to ignore the arterioles that also suffered injury; thus, we examined all small vessels with a diameter of 10–30 μm. We found a large number of discontinuities at the stria-like TJs in small vessels from multiple spinal segments (Fig. 3a, b), which have rarely been noticed in SCI previously. We defined discontinuities with a more than 70% reduction in fluorescence intensity and a length of 1.5–6 μm as gaps (Additional file 1: Fig. S4a). The density of gaps increased significantly within approximately 15 min, but no increase was observed in the spinal cords held 0 min post-SCI before being sacrificed; application of TTM for 4 h showed no protective effects against the formation of gaps (Fig. 3c–e). Notably, gaps were observed on almost all vessels in the harvested spinal segments (Fig. 3f–h). The expression of most TJ proteins remained stable 4 h post-SCI, suggesting that BSCB disruption early after SCI is not due to TJ degeneration. The gaps spread along the spinal cord may have been correlated with the spread of barrier leakage in the acute phase after SCI. Extensive gaps were observed on multiple spinal cord segments, including injury and penumbra area, at 15 min but not immediately post-SCI, indicating that this structural damage was a secondary change following primary physical strike.

### Pathological hemodynamic changes facilitate BSCB disruption early after SCI

**Fig. 4:**
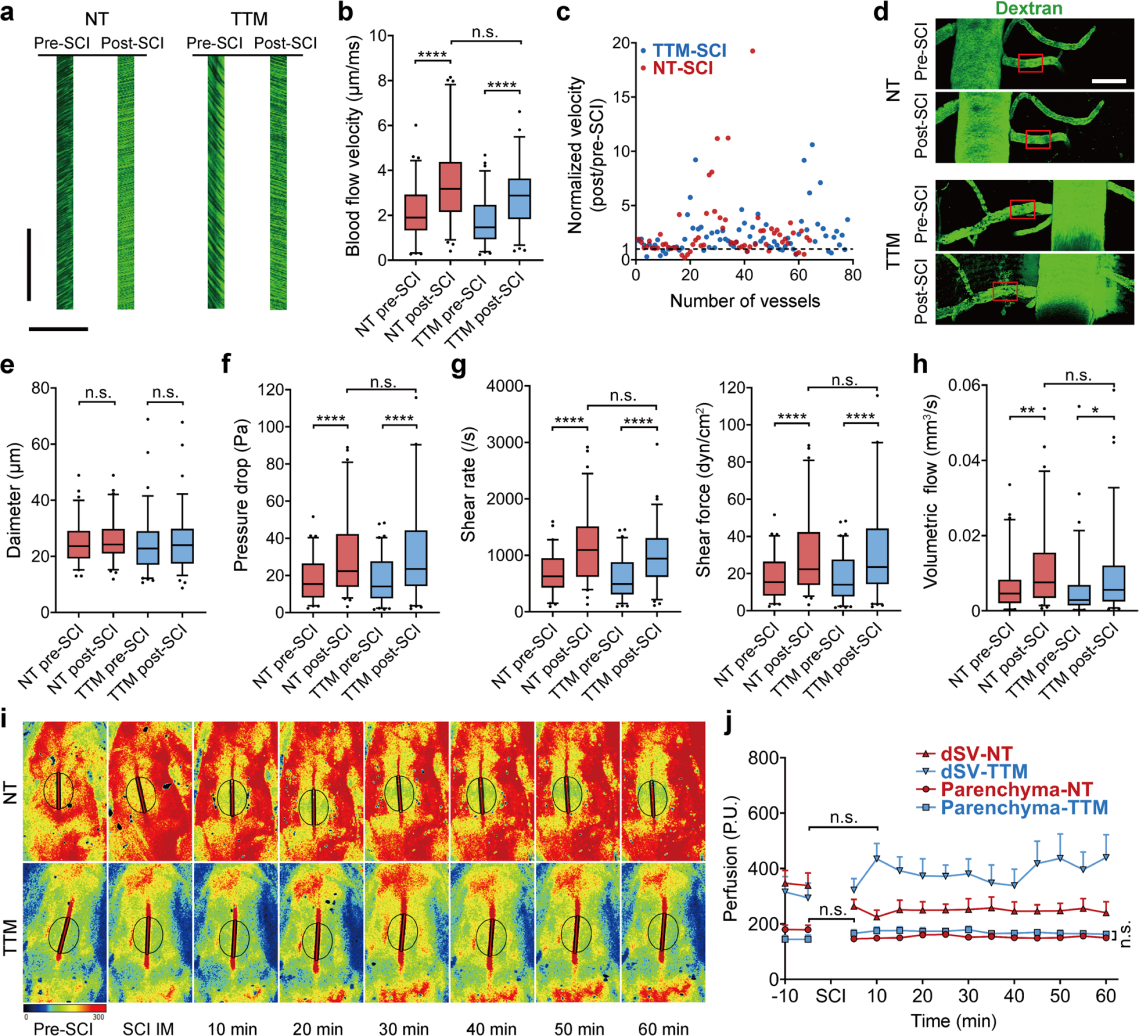
Pathological hemodynamic changes in dAVs post-SCI. **a** Representative kymographs of blood flow in dAVs obtained by two-photon microscopy using 150 kDa FITC-dextran pre- and post-SCI from the same vessel. The slope of the streak equals the velocity of each blood cell. Scale bar: vertical = 100 ms, horizontal = 200 μm. **b** Quantification of blood flow velocity in dAVs on the injured spinal segment within one-hour observation during NT or TTM (*n* = 8 mice, total 184 venules). The boundaries of the box indicate the 25th and 75th percentiles, and the whiskers indicate the 5th and 95th percentiles, the same below. **c** Blood flow velocity in each venule was normalized by self-comparison pre-SCI. **d** Representative maximum projection of the vessels in (**a**). The average diameter was calculated within the ROI (red frame). Scale bar = 100 μm. **e** Average diameter of dAVs in the ROI pre- and post-SCI with NT or TTM. **f** Pressure drop at dAVs in the ROIs pre- and post-SCI. The pressure drop in the ROI was calculated by the simplified Poiseuille formula and Radon-transform algorithm. A higher pressure drop indicated an increased pressure upstream in the ROI to maintain blood flow. **g** The shear rate and shear force in the dAVs during blood flow acceleration post-SCI were calculated according to the Newton inner friction law. The shear force is a frictional force exerted on the endothelium by blood flow. **h** The blood flow volume in dAVs within one hour post-SCI. **i**, **j** Blood perfusion in the dSV and parenchyma within an hour post-SCI with NT or TTM was measured by noncontact laser Doppler flowmetry. The redshift indicated more red blood cell movement (*n* = 6 mice). Data are presented as the mean ± SEM; *P < 0.05, **P < 0.01, ***P < 0.001, ****P < 0.0001; nested, one-way (**b**, **e**–**h**, **j**) and two-way (**j**) ANOVA

The mechanism by which primary damage leads to extensive formation of gaps post-SCI is unclear. Our in vivo imaging experiment revealed a significant acceleration of blood flow in dorsal venous vessels several minutes post-SCI (Additional file 2: Video S1 and Additional file 3: Video S2). Since the BSCB on the endothelium is directly exposed to blood flow, we next tested whether the previously unreported pathological hemodynamic changes could contribute to BSCB disruption. The blood flow in the venules is laminar flow and the least affected by breathing and heart pulsation [50]. The regions of interest (ROIs) were delineated on each dAV approximately 100–150 μm away from where they connected with the dSV, and hemodynamic parameters were calculated within these ROIs (Additional file 1: Fig. S4b, c). The blood flow velocity was calculated in vivo by line-scan imaging (Fig. 4a). Several minutes post-SCI, the velocity of blood flow in dAVs was significantly increased, and TTM had little effect on this increase in blood flow velocity (Fig. 4b). Acceleration of blood flow was observed in most dAVs on the imaged spinal segments (Fig. 4c), with no difference between the epicenter and penumbra (Additional file 1: Fig. S4d). The luminal diameter of each dAV in the ROI remained unaltered post-SCI (Fig. 4d, e; Additional file 1: Fig. S4e). Although the dSV diameter was altered shortly post-SCI, it returned to normal during blood flow acceleration (Additional file 1: Fig. S4f).

Pathological hemodynamic changes could exert abnormal physical forces to deteriorate the vessel wall [51]. We next calculated the parameters of the pathological hemodynamics exerted on the endothelium by Poiseuille’s law (Methods). The pressure gradient is the force that maintains blood flow, which also exerts an outward driving force on the lumen known as the transmural pressure to regulate the passive exchange of water-soluble substances through the BSCB [52]. Higher intravascular pressure indicates an increase in outward driving force on the injured vessels, promoting outward extravasation, hindering tissue fluid clearance, and aggravating tissue edema [53, 54]. During the pathological hemodynamic changes post-SCI, the pressure gradient in dAVs was markedly increased (Fig. 4f), indicating a significant increase in the driving force for BSCB leakage during this period. However, TTM did not have any significant effects on this increase in the pressure gradient (Fig. 4f).

Streaming blood also exerts a frictional force on the lumen of vessels called shear force. Physical shear force ranging from 10–20 dyn/cm^2^ is important for maintaining endothelial barrier integrity [55]. Previous studies have shown that pathological shear force can significantly deteriorate the morphological and functional properties of endothelial cells [56], disassemble paracellular junctions, and dysregulate barrier permeability [57]. In our study, the intravascular shear force and shear rate were estimated by simplified calculation according to Newton’s inner friction law [58]. Shear force was significantly increased in dAVs post-SCI, and this increase was not affected by TTM (Fig. 4g). It has been shown that shear forces higher than 40 dyn/cm^2^ dramatically alter junctional morphology and downregulate TJ proteins in vitro [56, 57]. During rapid and widespread BSCB disruption post-SCI, the shear force in more than a quarter of venules surpassed 40 dyn/cm^2^, indicating that this harmful level of frictional force affected a wide range of blood vessels.

Many studies have demonstrated reduced perfusion in the spinal cord as the injury progresses post-SCI [59–61], and the ischemia change likely affects the BSCB [62]. Therefore, we used the Radon transform algorithm to calculate volumetric blood flow in dAVs [63] and found that blood circumfluence in venules dramatically increased during the pathological hemodynamic change post-SCI (Fig. 4h). An increase in blood flow in downstream collecting venules may indicate an increase in blood supply in the injured spinal cord to protect against tissue ischemia or the formation of stationary clots in capillaries. However, the systemic mean blood pressure of the mice only had a slight and short increase approximately 10 min post-SCI (Additional file 1: Fig. S4g), indicating that this hemodynamic change in the spinal cord post-SCI did not originate from the heart but probably from the spinal cord. Because two-photon microscopy was unable to accurately analyze the complex blood flow in the dSV or microcirculation in the parenchyma, we used laser Doppler flowmetry to measure perfusion upstream and downstream of dAVs (Fig. 4i; Additional file 1: Fig. S4h). Perfusion in the parenchyma remained stable at an hour post-SCI, but perfusion in the dSV varied irregularly during TTM post-SCI (Fig. 4j). These data suggest no large variation in the blood perfusion of injured tissue during the pathological hemodynamic change, and the BSCB disruption may not be due to ischemia change in this period. Interestingly, blood flow stasis was repeatedly observed in small vessels after primary injury by in vivo two-photon microscopy (Additional file 2: Video S1). Some of this stasis was quickly eliminated, and recirculation occurred along with blood flow acceleration; moreover, blood components started to leak in the same time frame (Additional file 3: Video S2). Due to these pathological hemodynamic changes, the blood supply increased in venules and was maintained in the parenchyma during the early period of SCI; these changes seemed to exert an anti-ischemic or anti-blood stasis effect.

**Fig. 5:**
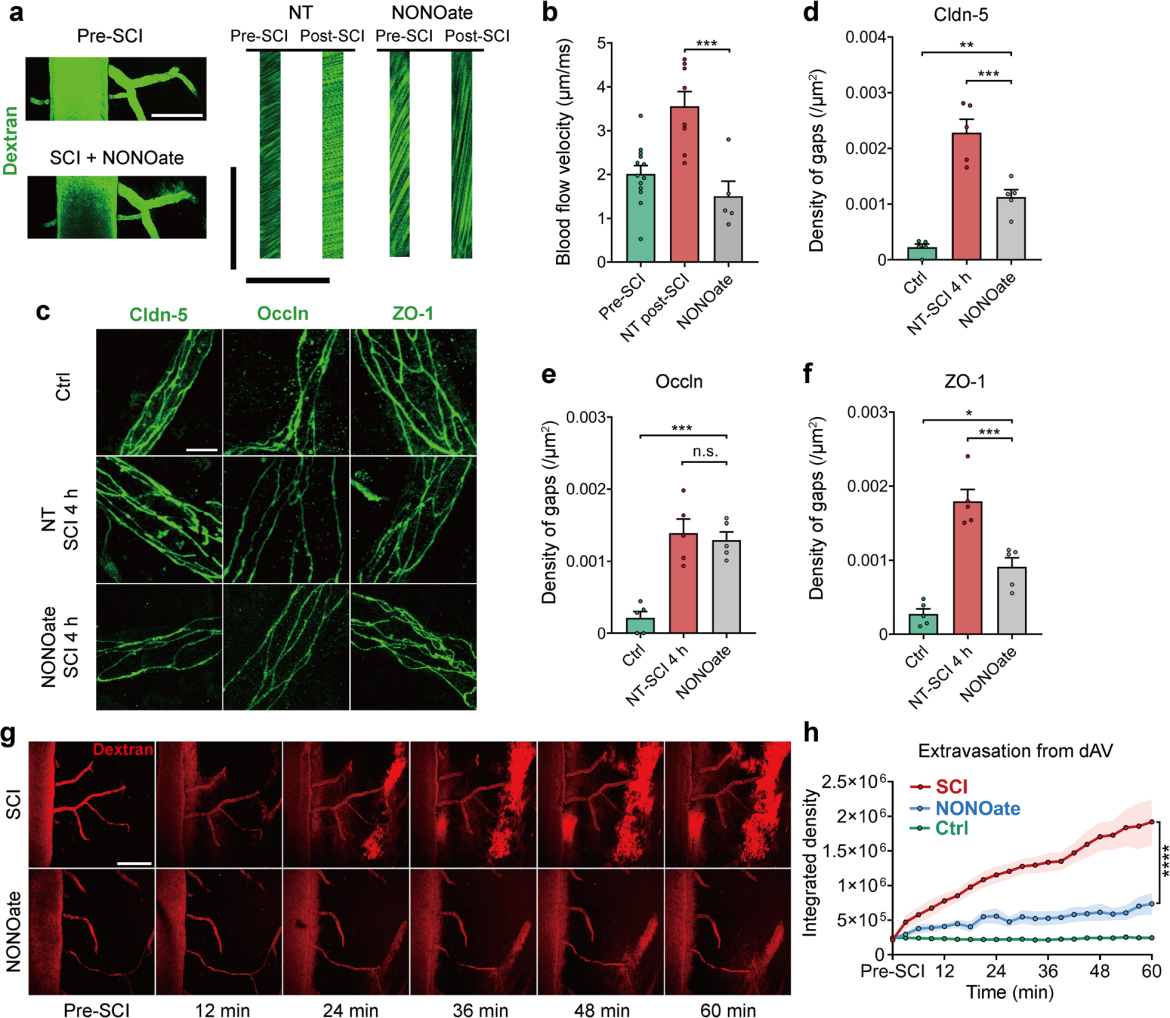
Alleviating pathological hemodynamic changes partially reduces gap formation on TJs and barrier leakage. **a** Vessel parameters were calculated following topical application of the NO donor NONOate post-SCI. Scale bar: vertical = 100 ms, horizontal = 200 μm. **b** Quantification of blood flow velocity in dAVs within one hour after NONOate application. Data from the NT pre/post-SCI group in Fig. 4b were replotted for comparison. The pre-SCI data were pooled (*n* = 8 and 5 mice, a total of 66 and 47 vessels). **c** Representative immunofluorescence images of TJs in small vessels treated with NONOate 4 h post-SCI. Scale bar = 5 μm. **d**–**f** Quantification of the gap density at TJs in the presence or absence of NONOate 4 h post-SCI. (**d**) claudin-5; (**e**) occludin; (**f**) ZO-1 (*n* = 5 mice, total 76, 80, and 136 vessels). **g** Time-lapse imaging of barrier leakage in the epicenter after acute SCI with the application of NONOate. Scale bar = 200 μm. **h** Quantification of the integrated fluorescence intensity of 40 kDa TRITC-dextran extravasated from the dAV in (**g**) (*n* = 8 mice, or *n* = 6 in the ctrl group). Data are presented as the mean ± SEM; *P < 0.05, ***P < 0.001, ****P < 0.0001; nested, one-way ANOVA (**b**, **d**–**f**) and two-way ANOVA (**h**)

To further confirm the role of pathological hemodynamic changes in BSCB disruption post-SCI, we employed a nitric oxide donor, diethylamine-NONOate, to alleviate blood flow acceleration via local vessel dilation [64]. After topical application of NONOate, the blood flow velocity in venules decreased dramatically post-SCI (Fig. 5a, b; Additional file 4: Video S3). Four hours after the application of NONOate, the density of gaps was reduced, as indicated by immunostaining of claudin-5 and ZO-1, but no changes in occludin were detected (Fig. 5c-f), suggesting that alleviating the pathological hemodynamic changes partly reduced gap formation post-SCI. The leakage of BSCB in the first hour after SCI was also reduced by applying NONOate, as monitored by two-photon microscopy (Fig. 5g, h). These results demonstrate that pathological hemodynamic changes play a critical role in rapid and widespread BSCB disruption post-SCI, probably by exerting increased pathological shear force and transmural force on the endothelium.

### Transmigration of hematogenous leukocytes disrupts the BSCB

It has been shown that the injury-induced immune response can cause the recruitment of leukocytes from the bloodstream to the injured spinal cord [3, 65]. Tricellulin, the only TJ protein that showed downregulated expression at 4 h post-SCI, forms the junctions between three endothelial cells, which are weak points for leukocyte transmigration [66]. Many histological studies have demonstrated that neutrophils and monocytes are the first inflammatory cells to appear in the parenchyma 4–6 h post-SCI [3, 65, 67]. Leukocytes can disrupt the BSCB by releasing metalloproteinases, the expression of which has been reported to be markedly increased at 8 h post-SCI [68, 69]. However, it remains undetermined whether leukocyte transmigration results from BSCB disruption or leukocyte transmigration actively breaches the BSCB in the early phase after SCI. Although previous studies have provided inconsistent evidence showing that leukocyte infiltration seems to occur later than BSCB disruption, we sought to determine whether leukocytes can breach the BSCB and correlate with gap formation in TJs shortly after primary SCI.

**Fig. 6:**
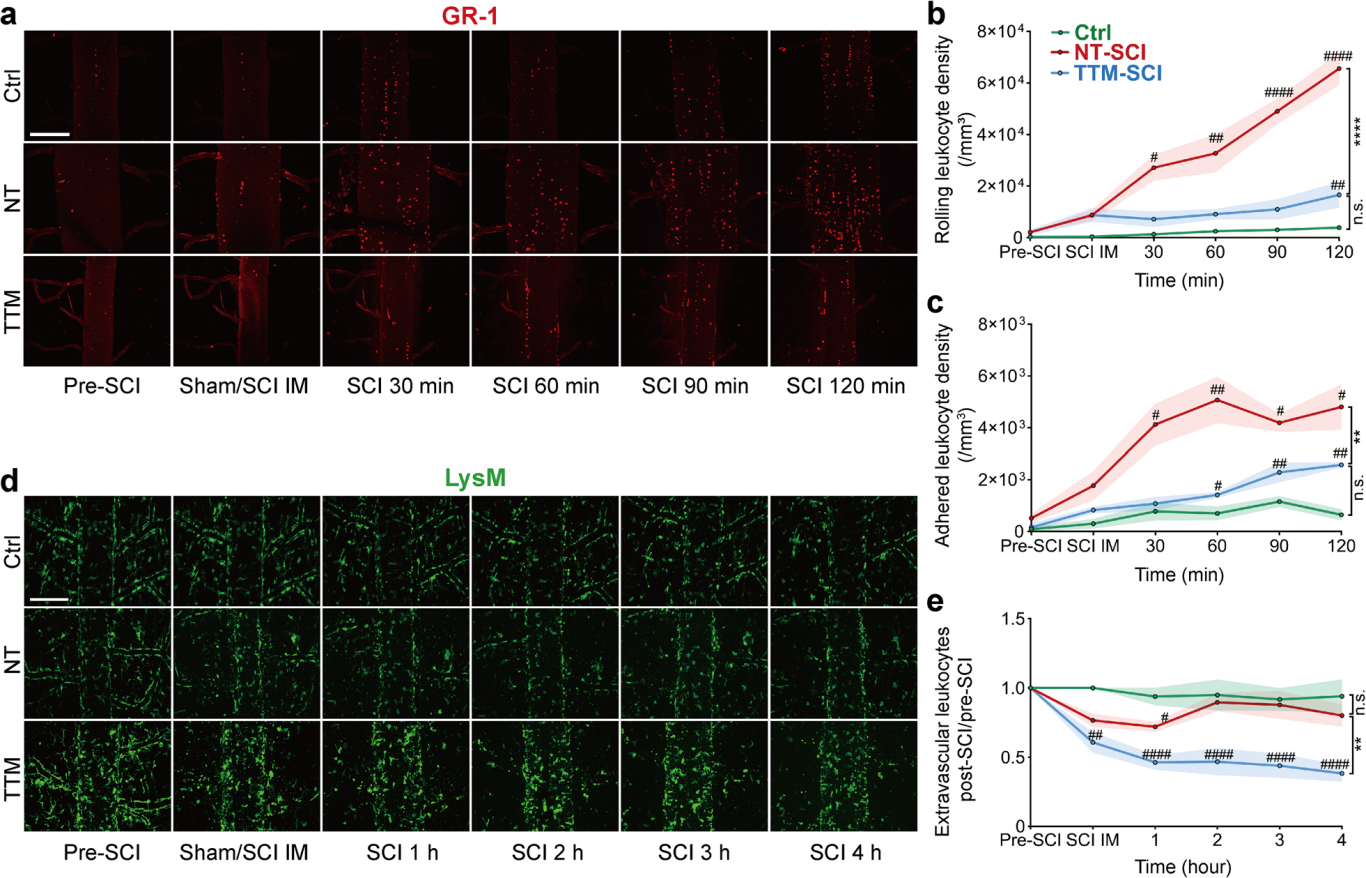
Hematogenous leukocytes actively transmigrate through the BSCB post-SCI. **a**–**c** Time-lapse imaging of activated PE-conjugated GR-1-labeled leukocytes in the dSV within two hours post-SCI. Scale bar = 200 μm. IM, immediate. This immediate time point for in vivo imaging was approximately 3–5 min post-SCI. Data were averaged from 3 longitudinally sequential ROIs from one spinal segment of each mouse. Quantification of the number of leukocytes rolling along (**b**) and adhered to (**c**) the lumen of the dSV within 2 h post-SCI (*n* = 6 mice). #*P* < 0.05, ##*P* < 0.01, ####*P* < 0.0001 by the Kruskal–Wallis test. **d**–**e** Time-lapse imaging of YFP-positive cells in *LysM*-YFP mice within 4 h post-SCI. Data from three ROIs on one spinal segment were averaged. Scale bar = 200 μm. (**e**) Quantification of YFP-positive cells in the extravascular area. The number of cells was normalized by that observed in the same ROI pre-SCI (*n* = 6 mice). Data are presented as the mean ± SEM; #*P* < 0.05, ##*P* < 0.01, ####*P* < 0.0001 by one-way ANOVA; **P < 0.01, ***P < 0.001 by two-way ANOVA; nested, one-way and two-way ANOVA (**b**, **c**, **e**)

To trace the infiltrating neutrophils and monocytes, a fluorescence-labeled GR-1 (Ly6G/C) antibody was administered via intravenous injection. Some GR-1-positive leukocytes started to roll along the wall of the veins within 3–5 min post-SCI (Fig. 6a), and more leukocytes rolled on the dSV than on venules (Additional file 5: Video S4). Many leukocytes quickly became deformed and connected with other leukocytes at the vascular junction between the dSV and spinal radial vein (Additional file 1: Fig. S5a), where the curved vessels are exposed to shear flow. Moreover, the anatomical position of the spinal radial vein is inconsistent and was occasionally captured, so we only collected data from the tubular dSV, which is arranged along the posterior median sulcus of the spinal cord. SCI induced a growing number of leukocytes rolling on and attaching to the endothelium likewise in the epicenter and penumbra (Additional file 1: Fig. S5b, c); this increase in the number of rolling and attached leukocytes was alleviated by TTM (Fig. 6b, c). Our results demonstrate that GR-1-positive leukocytes are quickly activated and initiate the transmigration process within 30 min post-SCI.

We next sought to investigate whether these leukocytes that adhered to the endothelium can produce gaps in TJs when BSCB disruption is extended while TJ expression remains unchanged. Since most GR-1-labeled leukocytes disappeared in the parenchyma, *LysM*-YFP transgenic mice were used to achieve stable imaging of leukocytes (*LysM*-YFP positive) that traversed the BSCB (Fig. 6d). Some cells with weak YFP expression outside of blood vessels in *LysM*-YFP mice are likely microglia [70, 71], which also carry lysozymes and are very difficult to distinguish from transmigrated macrophages in vivo [72, 73]. After SCI, many YFP-positive cells adhered to the luminal walls of vessels in the epicenter (Additional file 1: Fig. S5d, e), and no difference in the number of extravascular YFP-positive cells was detected in the injured spinal segment among the three groups, either in the epicenter or penumbra (Additional file 1: Fig. S5f). Curiously, the number of extravascular YFP-positive cells was slightly reduced and returned to the normal level at 2 h post-SCI during NT, and the number of extravascular YFP-positive cells decreased during TTM in the 4-h observation period post-SCI (Fig. 6e). This difference probably denotes different transmigration rates of leukocytes in the NT and TTM groups.

**Fig. 7:**
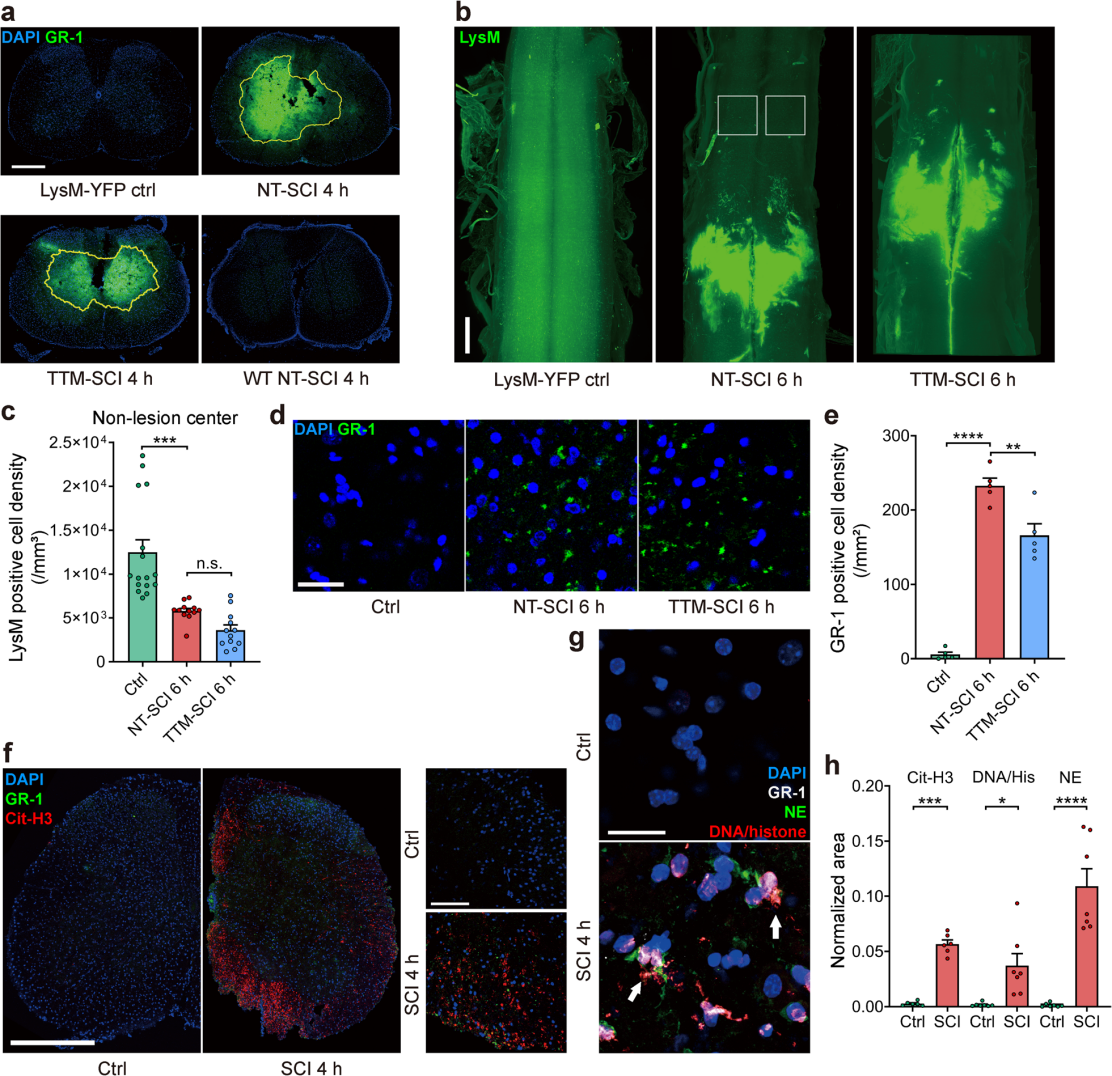
Leukocytes migrate into the parenchyma. **a** A lesion core formed in the epicenter (yellow frame) via aggregation of YFP-positive cells in the gray matter. Sections of the spinal cord were harvested from *LysM*-YFP mice 4 h post-SCI (*n* = 3 independent experiments). WT: wild-type mice. Scale bar = 500 μm. **b**,** c** Whole-mount images of the spinal cords of *LysM*-YFP mice 6 h post-SCI. (**b**) Representative whole-mount images (displayed with a gamma correction of 1.5). Scale bar = 500 μm. (**c**) Quantification of the density of YFP-positive cells in the penumbra area. These cells were counted in 500^3^ μm.^3^ regions (white frame) 1 mm away from the lesion core in (**b**) (*n* = 3 to 4 mice). **d** Representative images of GR-1-positive leukocytes by immunostaining 6 h post-SCI. Scale bar = 25 μm. **e** Quantification of the GR-1-positive leukocyte density in multiple spinal segments 6 h post-SCI in (**d**) (*n* = 5 mice). **f** GR-1-labeled neutrophils (green) released nuclear citrullinated histone H3 (cit-H3, red) into nearby tissue 4 h post-SCI (*n* = 6 mice). Scale bar: left = 500 μm, right = 100 μm. **g** GR-1-labeled neutrophils (gray) released nuclear DNA/histone complex (red) and cytoplasmic neutrophil elastase (NE, green) into nearby tissue 4 h post-SCI (*n* = 7 mice). Scale bar = 25 μm. **h** Normalized area of the neutrophils released cit-H3, DNA/histone and NE in the neural parenchyma in (**f**, **g**). The area was normalized by the size of the pictures. Data are presented as the mean ± SEM; **P < 0.01, ****P < 0.0001; nested, one-way ANOVA (**c**, **e**, **h**)

Previous studies have demonstrated that leukocytes transmigrate into the injured parenchyma after SCI [1, 3], and our data have shown that leukocyte transmigration is initiated within 30 min post-SCI (Fig. 6). However, it was interesting that the number of extravascular YFP-positive cells was not increased during 4 h of observation with NT or TTM (Fig. 6e; Additional file 1: Fig. S5f). We speculated that leukocytes might migrate beyond the detection range of two-photon microscopy or disappear through other mechanisms. Therefore, we examined transverse spinal cord sections from *LysM*-YFP mice 4 h post-SCI and found that a large number of YFP-positive puncta aggregated in the gray matter in the epicenter and formed a lesion core (Fig. 7a). Next, light-sheet microscopy was utilized to image whole spinal segments from *LysM*-YFP mice after uDISCO tissue clearing. There was a decrease in the number of YFP-positive cells in the penumbra area at 6 h post-SCI (Fig. 7c), which is consistent with our previous in vivo imaging results (Fig. 6e). In comparison, a large number of YFP-positive puncta aggregated in the lesion core (Fig. 7b), probably formed by the YFP-positive cells swarming into the gray matter at the epicenter.

Many studies have reported that neutrophils are short-lived in inflammatory tissue [67]. Since the Ly6G antibody did not work well for immunostaining, the GR-1 antibody was used to label neutrophils and monocytes in the transection slices (Fig. 7d; Additional file 1: Fig. S6a). Many GR-1-positive cells infiltrated the epicenter at 6 h post-SCI, and the number of these cells was reduced under TTM (Fig. 7e; Additional file 1: Fig. S6b). Interestingly, neutrophils that arrived in the parenchyma seemed to undergo netosis, characterized by the release of DNA-histone complexes, citrullinated histone H3, and neutrophil elastase into nearby tissue, as demonstrated by immunostaining at 4 h after SCI (Fig. 7f–h; Additional file 1: Fig. S6c). These components released from neutrophils could be engulfed by other inflammatory cells and cause further damage [74]. Our results showed that leukocytes swarm to the gray matter in the epicenter after crossing the BSCB within 4 h post-SCI. The leukocytes further transmigrate and are lost in the parenchyma, which could partially explain why there was no increase in the number of leukocytes in the parenchyma within 4 h post-SCI. In summary, the leukocytes started to roll along the vessels within minutes, initiated the transmigration process within 30 min post-SCI (Fig. 6), underwent netosis in the neural parenchyma, or arrived at the lesion core in the gray matter within 4 h (Fig. 7). These data suggest that the leukocytes have traversed the BSCB into the neural parenchyma in the early period of SCI (much earlier than 4 h), coinciding with BSCB disruption spreading.

**Fig. 8:**
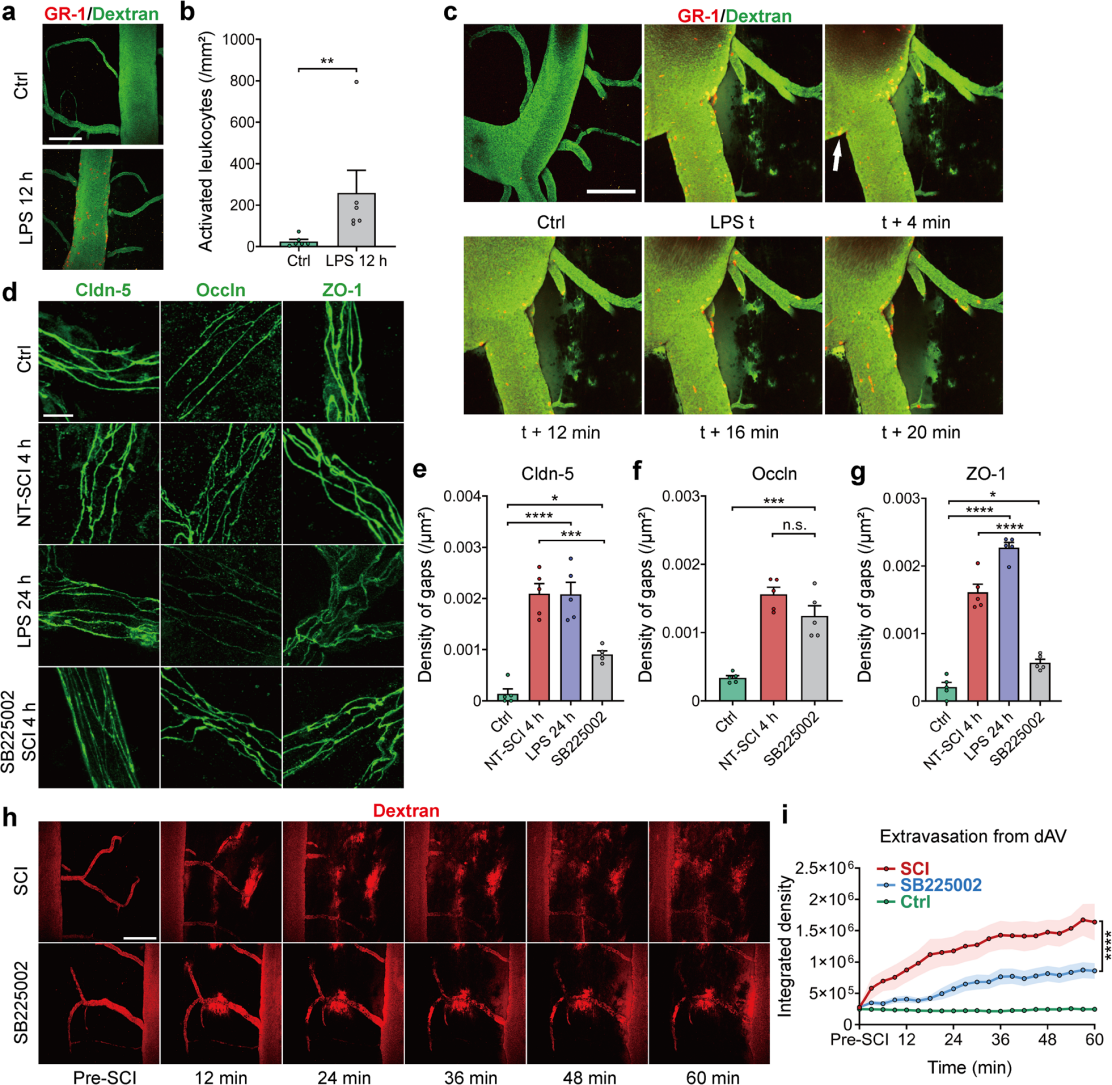
Leukocyte transmigration actively contributes to BSCB disruption. **a** Representative images of LPS-induced leukocytes (labeled by Alexa Fluor 700-conjugated GR-1) adhered to the lumen of vessels (labeled by 150 kDa FITC-dextran). Scale bar = 200 μm. **b** Quantification of activated leukocytes in the lumen of the dSV 12 h after LPS injection (*n* = 6 mice). **c** Representative images of BSCB leakage (white arrow) induced by LPS-activated leukocytes at 12 h (*n* = 4 independent experiments). Scale bar = 200 μm. **d** Representative immunofluorescence images of TJs in small vessels 24 h after LPS injection or application of SB225002 4 h post-SCI. Scale bar = 5 μm. **e**–**g** Quantification of gap density at TJs in (**d**). (**e**) Claudin-5; (**f**) occludin; (**g**) ZO-1 (*n* = 5 mice, a total of 114, 121, 118, 167 vessels). **h** Time-lapse imaging of barrier leakage in the epicenter after acute SCI with SB225002 application. Scale bar = 200 μm. **i** Quantification of the integrated fluorescence intensity of 40 kDa TRITC-dextran extravasated from the dAV in (**h**). Data from the ctrl group were replotted from Fig. 5h (*n* = 9 mice, or *n* = 6 in the ctrl group). Data are presented as the mean ± SEM; *P < 0.05, **P < 0.01, ****P < 0.0001; nested, the Mann–Whitney test (**b**); one-way ANOVA (**e**–**g**); general estimated Equation (**i**)

To further explore whether the infiltration of leukocytes can lead to BSCB leakage, colibacillus lipopolysaccharide (LPS) was intraperitoneally injected into mice to induce leukocyte infiltration [75]. The in vivo imaging data showed that some GR-1-positive leukocytes adhered to the luminal wall after LPS injection (Fig. 8a, b; Additional file 1: Fig. S6d). Dextran leakage began ~ 10 min after the leukocytes adhered to the vessel wall (Fig. 8c), illustrating that the BSCB leakage position coincides with the leukocyte adherence sites. To further confirm that the infiltration of leukocytes can induce gap formation in TJs in the early period of SCI, the C-X-C chemokine receptor type 2 (CXCR2) antagonist SB225002 was administered post-SCI to inhibit interleukin-8-induced neutrophil infiltration [76]. Application of SB225002 after SCI significantly decreased the formation of gaps, while LPS application resulted in a marked increase in the formation of gaps in TJs (Fig. 8d–g). These results show that leukocyte infiltration induced by LPS, which is similar to SCI, can actively induce gap formation, while inhibition of leukocyte diapedesis can prevent gap formation in the BSCB after SCI. The leakage of the BSCB in the first hour after SCI also decreased with the administration of SB225002 (Fig. 8h, i), indicating that inhibiting leukocyte transmigration can reduce BSCB leakage in early SCI. In summary, these results demonstrate that leukocyte transmigration actively induces extensive gap formation and BSCB disruption in the early phase of SCI.

## Discussion

SCI is a relatively common injury in the clinic, and most traumatic SCI cases result from physical trauma [2, 77]. Primary mechanical injury initiates a complex secondary injury cascade resulting in continuous neuronal and glial death [2]. Protecting neurons from continuous and widespread secondary injury in minutes to weeks [78] should be a priority in the treatment of SCI. The BSCB maintains the immune isolation of the spinal cord, and its disruption is a major exacerbating factor in secondary injury [3]; thus, protecting or re-establishing BSCB function is critical to alleviating the amplifying secondary injury. To further examine BSCB disruption in SCI, we combined in vivo imaging and postmortem analysis to investigate the rapid and widespread BSCB disruption in the early phase post-SCI and found that BSCB disruption occurred rapidly post-SCI and spread farther to adjacent spinal segments. Many gaps appeared on TJs along small vessels at multiple spinal segments, which likely accounts for widespread paracellular leakage of the BSCB. Abnormal shear force and transmural pressure induced by pathological hemodynamic changes contribute to BSCB disruption in the early phase post-SCI. Leukocyte transmigration, which occurs to a significant degree within 30 min after SCI, also actively contributes to BSCB disruption. TTM did not have any significant protective effects against BSCB disruption in the early period of SCI. Our results demonstrated that pathological hemodynamic changes and leukocyte transmigration participate in rapid and widespread paracellular BSCB disruption post-SCI.

BSCB disruption was detected within several minutes after SCI (Fig. 1b, c), which is consistent with the findings of other rodent studies that BSCB disruption occurs within 5–15 min [12, 79]. BSCB disruption gradually spreads to adjacent spinal segments (Fig. 1b, f), which is in agreement with previous studies [12], and possibly even extends along the entire length of the spinal cord in the worst cases [1, 14]. The conventional view suggests that physical trauma causes BSCB disruption post-SCI through shear injury or tissue strain [1, 12]. This theory cannot clearly explain how the relatively confined primary physical injury leads to widespread BSCB disruption after SCI. Furthermore, if the primary mechanical impact were to cause widespread vessel rupture, the leakage of BSCB would be synchronized in a short time, rather than gradually spread along the spinal cord [80, 81]. Other factors must exacerbate the damage to the BSCB following primary mechanical injury.

Secondary injury is characterized by the expansion of tissue damage, including inflammation-induced apoptosis of adjacent cells [3]. It has been shown that inflammatory factors can downregulate the expression of TJ proteins during secondary injury [82] between 8 and 24 h post-SCI [9–11, 83]. Moreover, a decrease in TJ protein expression usually indicates that BSCB disruption is more severe than just paracellular leakage and involves processes such as endothelial cell degeneration, during which whole cells are lost [8]. Our results demonstrated that the membrane expression of major TJ proteins was not altered at 4 h post-SCI (Fig. 2 and Additional file 1: Fig. S2), while leakage of the BSCB started to spread along the spinal cord several minutes post-SCI (Fig. 1). Many gaps appeared in TJs at noncapillary small vessels within 15 min post-SCI (Fig. 3c). Theoretically, even small gaps can disrupt the continuity of the BSCB and allow for paracellular leakage[42]. A study by Baluk et al. [84] showed that endothelial gaps contribute to plasma leakage in rodent trachea and that most of the leakage occurs in postcapillary venules with more endothelial gaps. Sporadic gaps in TJs were detected at intact vessels in our study (Fig. 3 and Additional file 1: Fig. S3), which were probably due to the loose organization of TJs in venules [42, 85] or ice crystal formation during tissue preparation [86]. Our findings that gaps are widely present in multiple spinal segments 15 min post-SCI but not immediately post-SCI suggest that these gaps are unlikely to be formed via mechanical rupture but instead due to secondary injury.

The extensiveness of gap formation indicates that secondary factors that breakdown the BSCB may be spatially widespread. We observed pathological hemodynamic changes in venous vessels several minutes post-SCI (Fig. 4a–h; Additional file 2: Video S1 and Additional file 3: Video S2); these changes may affect the barrier exposed to blood flow by exerting higher physical forces, such as shear force. Some in vitro results have shown that pathological shear force decreases the expression of TJ proteins, alters junctional morphology (> 40 dyn/cm^2^) [57, 58], induces translocation (> 20 dyn/cm^2^) [87], and increases vascular permeability in response to inflammation (> 28 dyn/cm^2^) [88]. It should be noted that the transmural pressure generated by the pressure gradient exerted on the vessel endothelium was underestimated (Fig. 4f) because the actual length of the vessels was much greater than the length used for calculation in the formula (Additional file 1: Fig. S4b). In our study, partially alleviating pathological blood flow acceleration reduced the number of gaps in TJs post-SCI (Fig. 5d–f) and barrier leakage (Fig. 5g, h), further demonstrating that pathological hemodynamic changes can cause BSCB disruption after SCI. Unfortunately, the origin of these pathological hemodynamic changes remain unclear. The systemic blood pressure only slightly increased approximately 10 min post-SCI (Additional file 1: Fig. S4g), indicating that these hemodynamic changes were not due to cardiac output. The pathological hemodynamic changes may not arise from the venous system because venous vessels are collecting vessels with little regulatory flexibility due to a lack of smooth muscle [53]. However, the vessels can dilate when blood flow exerts a higher shear force, termed flow-induced vasodilation [89]. In our experiments, the venules did not dilate when the shear force increased (Fig. 4e, g; Additional file 1: Fig. S4e), which implies that these venules might also show a trend of vasoconstriction against passive flow-induced vasodilation. These pathological hemodynamic changes may likely originate from the arteries due to blood flow autoregulation in the spinal cord [90] but still require further investigation.

During the pathological hemodynamic changes, blood perfusion increased at venules and was preserved in the parenchyma, at least in the first hour post-SCI (Fig. 4h–j). Interestingly, many studies have demonstrated a reduction in perfusion in the spinal cord post-SCI [59, 60]. This discrepancy in blood supply in the early phase after SCI might be due to differences in the methods used for blood perfusion calculation. Some microangiography or fluorescent tracer studies have shown that perfusion is preserved in the white matter for 24 h and in the gray matter for an hour post-SCI [91]. Senter et al. [92] observed an augmentation of blood perfusion maintained 1 to 2 h before the onset of ischemia and speculated that this phenomenon came from the autoregulation of the spinal cord. This finding may be consistent with the pathological hemodynamic changes observed in our study. Moreover, the pathological hemodynamic changes after SCI do not conflict with the occurrence of ischemia but could help explain the origin of ischemia in secondary injury. Pathological hemodynamic changes can weaken the BSCB, induce more outward filtration and further promote swelling and edema [53]. Since the spinal cord is located in a confined space framed by bone and dura, edema-induced position shifts of the spinal cord tissue can increase local pressure on the parenchyma, thus reducing blood flow into tissue and exacerbating ischemia [31, 53].

Thus far, it is undetermined whether leukocyte infiltration leads to early BSCB disruption after SCI. Some in vitro evidence suggests that leukocyte transmigration can result in barrier leakage [35, 93] and the formation of intracellular gaps between cultured endothelial cells [94], in adherens junctions and TJs [66, 95]. Our results showed that leukocytes actively adhered to the BSCB after SCI and could breach the BSCB by producing gaps in TJs (Figs. 6 and 8). Inhibition of neutrophil infiltration with a CXCR2 antagonist decreased the formation of gaps and barrier leakage post-SCI (Fig. 8d–i). In addition, similar to SCI, injection of LPS induced leukocyte transmigration and the formation of gaps in small vessels (Fig. 8d–g). LPS-activated leukocytes induced leakage 10 min after adhering to the endothelium (Fig. 8c). These results illustrate that infiltrating leukocytes can actively breach the BSCB by producing gaps in TJs and result in barrier leakage in the early period after SCI.

After SCI, leukocytes are activated and start to transmigrate rapidly, as indicated by rolling and adherence to the vascular endothelium (Fig. 6a–c). Some in vivo studies on muscular vessels have shown that neutrophils take ~ 6 min to traverse the endothelium and ~ 40 min to traverse the basal membrane [95, 96]. However, there is an apparent time lag considering that many histological studies have shown that neutrophils and monocytes are the first peripheral inflammatory cells detected in the parenchyma 4–6 h after SCI [3, 97, 98]. In our data, LysM-positive leukocytes further migrated into the gray matter after arriving in the parenchyma, forming a lesion core within 4 h post-SCI (Fig. 6d, e and Fig. 7a–c). It has been shown that this lesion core is mainly formed by blood-derived myeloid cells but few microglia during the first couple of days after SCI in LysM transgenic mice [99]. In particular, neutrophils underwent netosis after arriving in the parenchyma, as indicated by the release of nuclear and plasma components (Fig. 7f, g; Additional file 1: Fig. S6c), which could be engulfed by other cells [74, 100]. These results indicate that leukocytes traverse the BSCB and arrive in the neural parenchyma much earlier in SCI than previously thought. Furthermore, the role of neutrophils in SCI remains controversial [73, 101, 102]. Our finding that neutrophils release marker proteins such as the DNA/histone complex, citrullinated histone H3, and neutrophil elastase into the parenchyma in SCI is very similar to the neutrophil extravascular traps observed in other autoimmune diseases [103, 104]. Some studies have shown that intracerebral injection of neutrophil elastase can cause endothelial swelling and focal necrosis of blood vessels in the brain [65, 105]. A recent study on cerebral stroke found that neutrophils exhibit similar behavior, further damaging the blood–brain barrier and impairing vascular remodeling [106]. Future studies exploring this neutrophil behavior in the injured spinal cord may provide further insight into the mechanism of SCI.

Since inflammatory substances can leak through the BSCB to promote secondary injury, therapeutic methods applied later after SCI, such as immunosuppressive agents, often have few effects. Thus, an efficient method to protect the BSCB after SCI is desperately needed, especially in the acute period. Some studies have observed that TTM (33 °C for 4 h) could protect the barrier and limit its permeability after brain trauma [18–22, 107], but the underlying mechanism is unclear. However, TTM under similar conditions did not show any significant protective effects against BSCB disruption in early SCI in our study, except for partially reducing leukocyte transmigration. Nevertheless, applying TTM during such an early period would be extremely difficult in actual operation. Applying TTM earlier will have better protective effects against BSCB disruption, which could pervade the spinal cord and exacerbate secondary injury. However, TTM under ideal conditions remains insufficient to protect the BSCB in SCI. In addition, TTM with more prolonged time or lower temperature could have more complications, which need further intensive care and might hinder its clinical effects.

The additional complications introduced by TTM, such as platelet and coagulation dysfunction [108, 109], could interfere with the hemostasis process, exacerbating microhemorrhage and blood component extravasation. Despite TTM decreasing the rolling, adherence, and infiltration of leukocytes, which seems to have an anti-inflammatory effect and should partially block BSCB disruption, our results showed that TTM did not produce an overall therapeutic effect on BSCB disruption. The extravasation from venules was visually higher in the TTM group after SCI, and membrane expression of ZO-1 remained unaltered during BSCB leakage but decreased in TTM-treated animals. These discrepancies suggest that the side effects of TTM are entangled with its therapeutic effects, which means TTM itself does not have a neat therapeutic effect on the BSCB in SCI. Combined therapies that could reduce TTM complications while retain inhibition of leukocyte transmigration may be beneficial in SCI treatment, but more trials are needed.

## Conclusions

Our study demonstrates that extensive gap formation in TJs is the main pathological manifestation of BSCB disruption in the early phase after SCI. Pathological hemodynamic changes and leukocyte transmigration play a critical role in widespread gap formation in TJs in this period. Since widespread BSCB disruption is mainly generated in secondary injury, this hints at a potential time window for clinical interventions to alleviate BSCB leakage and inhibit the continuous spread of BSCB disruption. These results improve our understanding of the role of BSCB disruption in the acute phase after SCI and can guide the development of new therapeutic approaches for attenuating BSCB disruption to ultimately improve SCI patient outcomes in the clinic.

## Supplementary Information

**Additional file 1: Fig. S1.** **a** A homemade imaging installation. The chamber was cemented by dental cement after using the clamps to hold the vertebral column of the mouse. This installation was used for rapid imaging of the injury site before and after SCI. **b** A schematic timeline of in vivo imaging. **c** Temporal explanation of the steps of BSCB disruption after SCI. **d** Representative maximum projection about the extravasation of 40kDa TRITC-dextran 30 min post-SCI. The frames were manually drawn to quantify the extravascular fluorescence density leaked from dSVor dAVin ImageJ. **e** Quantification of fluorescence intensity extravasated from dSV in the epicenter from Fig. 1c. Data are the mean ± SEM, n.s. no significance; nested, general estimated equation. f Representative immunohisto-chemistry images of serum immunoglobulinextravasation near the epicenter. A cystappeared 4 hours post-SCI. Scale bar = 1 mm. **g** The membrane component obtained by the Mem-PER kitwas detected by ATPase and β-actin antibodies. ATPase is a pump of hydrogen and potassium ions located on the membrane and was used as a membrane protein loading control; the β-actin was used as a plasma protein loading control. The loading lysis was diluted to 10-3 μg/μl by the optimized detection concentration for ATPase. **Fig. S2.** Representing graph view about chemiluminescence intensity of junctional proteins measured by automated capillary western assay 4 hours post-SCI. **a** claudin-5; **b** occludin; **c** ZO-1; **d** tricellulin; **e** CD31; **f** ATPase; **g** vinculin. Vinculin was used as a loading control for ZO-1, and ATPase was used as a loading control for the other proteins. The expression of proteins was calculated automatically by the peak-area methodin Compass software. **Fig. S3.** Gap formation in TJs at non-capillaries small vessels post-SCI. **a**–**c** Representative images of claudin-5, occludinand ZO-1 at non-capillary small vessels post-SCI. The same images were merged with CD31. Scale bar = 25 μm. The gaps in TJs were detected on these vessels. TJs on small vessels at different time-point post-SCI with NT or TTM.; cldn-5 on spinal cord 15, 30, 60 minutes post-SCI, total 58, 63, 68 vessels respectively). TJs at small vessels manipulated with NONOate 4 hours post-SCI..TJs at the small vessels 24 hours after LPS injection or manipulated with CXCR2 antagonist SB225002 4 hours post-SCI. The gap was measured by the line intensity methodin LasX software. The discontinuity characterized as a more than 70% decrease in fluorescence intensity< 0.05, nested, one-way ANOVA; the Mann–Whitney test. **Fig. S5. a** Representative images about the PE-conjugated GR-1 labeled leukocytes were activated at the vascular junction between the dSV and spinal radial vein post-SCI under a CCD cameraand two-photon microscope. Scale bar: left = 250 μm, right = 100 μm. Many leukocytes quickly got deformed or connected to other leukocytes. **b** Density of leukocytes rolling on the lumen of dSV in epicenter and penumbra from. c Density of leukocytes adhered on the lumen of dSV in epicenter and penumbra from Fig. 6c. **d**, **e** Images about the most extreme case of YFP-positive cells adhered to the lumen. The ROI under CCD cameraand under two photon microscope. The extravascular LysM positive cells decreased over time, and the outline of dAVs gradually became invisible. Scale bar: left = 150, right = 200 μm. f YFP labeled leukocytes and/or microglia in epicenter and penumbra from Fig. 6e. **g**, **h** Density of leukocytes rolling or adhering on the lumen of dSV. i Density of YFP labeled leukocytes and/or microglia in the extravascular area. The vasoactive agent NONOate does not have an apparent effect on leukocyte transmigration. Data are the mean ± SEM; n.s. no significance, ^#^*P* < 0.05, ^##^*P* < 0.01, ^####^*P* < 0.0001. nested, Kruskal–Wallis test and two-way ANOVA. **Fig. S6.** **a** Representative images of GR-1 positive leukocyteson cross-sections 6 hours post-SCI. Scale bar = 500 μm. **b** Quantification of GR-1 positive leukocytes in multiple spinal segments 6 hours post-SCI. The value was adjusted by the number of DAPI positive nuclei. c A representative image about GR-1 labeled neutrophilsreleased nuclear DNA/histone complexand cytoplasmic neutrophils elastaseinto nearby tissue 4 hours post-SCI. The extracellular neutrophils elastase formed a spiky-like structure, and the cell-free DNA/histone formed a cloudy-like structure. Scale bar = 25 μm. d Representative images about AlexaFluor 700 conjugated GR-1 labeled leukocytesactivated after LPS injection. The plasma was labeled by 40 kDa FITC-dextran. The leakage related to leukocytes transmigration was less at tubular dSVthan at vascular junction between the dSV and spinal radial vein in. Scale bar: left = 250 μm, right = 100 μm. Data are the mean ± SEM; *P < 0.05, *P < 0.0001. nested, one-way ANOVA. **Table S1. **Antibodies or fluorescence materials used in this study.

**Additional file 2: Video S1.** Blood flow stasis or blood clots appears at dAVs in the epicenter immediately post-SCI. Time-lapse fluorescence microscopy was captured under a CCD camera by intravenous injection of 150 kDa FTIC-dextran. Some blood clots could form to hinder the blood flow. This phenomenon usually lasts less than 5 minutes and could be repeatedly observed in independent experiments.

**Additional file 3: Video S2.** Blood flow stasis is eliminated by blood flow acceleration in dAVs shortly post-SCI. The ROI in this video was the same region as shown in Additional file 2: Video 1 several minutes later. The stasis or blood clot was quickly eliminated, and vessel recirculation occurred along with blood flow acceleration. The pathological blood acceleration arose, and the blood flow stasis or blood clots were eliminated. The blood components started to leak in the same time frame.

**Additional file 4: Video S3.** Blood flow acceleration post-SCI was partly slowed down by topical application of NONOate. Time-lapse fluorescence microscopy was captured by CCD camera by intravenous injection of 150 kDa FTIC-dextran.

**Additional file 5: Video S4.** Activated leukocytes roll and adhere on the vessel endothelium. Time-lapse fluorescence microscopy showed by intravenous injection of PE-conjugated GR-1to label leukocytes and 150 kDa FTIC-dextran to label plasma. Some leukocytes have already transferred through the BSCB into parenchyma or attached to the dura.

**Additional file 6.** Statistical test results.

**Additional file 7.** Full images of western blots presented in main figures.

## Data Availability

All data generated or analyzed during this study are included in the published article.

## Abbreviations

SCI: Spinal cord injury
BSCB: Blood–spinal cord barrier
TJ: Tight junction
TTM: Target temperature management
NT: Normothermia
IM: Immediately
LPS: Lipopolysaccharide
ZO: Zonula occludens
CXCR2: C-X-C chemokine receptor type 2
