# Supplementary material for: Pathological hemodynamic changes and leukocyte transmigration disrupt the blood–spinal cord barrier after spinal cord injury
Source: J Neuroinflammation. 2023 May 20;20:118. doi: 10.1186/s12974-023-02787-w (PMC10200062; doi:10.1186/s12974-023-02787-w)
Supplement: Supplementary file 1 — Additional file 1: Fig. S1. a A homemade imaging installation. The chamber was cemented by dental cement after using the clamps to hold the vertebral column of the mouse. This installation was used for rapid imaging of the injury site before and after SCI. b A schematic timeline of in vivo imaging. c Temporal explanation of the steps of BSCB disruption after SCI. d Representative maximum projection about the extravasation of 40kDa TRITC-dextran 30 min post-SCI. The frames were manually drawn to quantify the extravascular fluorescence density leaked from dSVor dAVin ImageJ. e Quantification of fluorescence intensity extravasated from dSV in the epicenter from Fig. 1c. Data are the mean ± SEM, n.s. no significance; nested, general estimated equation. f Representative immunohisto-chemistry images of serum immunoglobulinextravasation near the epicenter. A cystappeared 4 hours post-SCI. Scale bar = 1 mm. g The membrane component obtained by the Mem-PER kitwas detected by ATPase and β-actin antibodies. ATPase is a pump of hydrogen and potassium ions located on the membrane and was used as a membrane protein loading control; the β-actin was used as a plasma protein loading control. The loading lysis was diluted to 10-3 μg/μl by the optimized detection concentration for ATPase. Fig. S2. Representing graph view about chemiluminescence intensity of junctional proteins measured by automated capillary western assay 4 hours post-SCI. a claudin-5; b occludin; c ZO-1; d tricellulin; e CD31; f ATPase; g vinculin. Vinculin was used as a loading control for ZO-1, and ATPase was used as a loading control for the other proteins. The expression of proteins was calculated automatically by the peak-area methodin Compass software. Fig. S3. Gap formation in TJs at non-capillaries small vessels post-SCI. a–c Representative images of claudin-5, occludinand ZO-1 at non-capillary small vessels post-SCI. The same images were merged with CD31. Scale bar = 25 μm. The gaps in TJs were detected on t [file 12974_2023_2787_MOESM1_ESM.pdf]

Pathological hemodynamic changes and leukocyte transmigration disrupt the blood–spinal cord barrier after spinal cord injury

Rubing Zhou[1,2], Junzhao Li[2], Zhengyang Chen[1], Ruideng Wang[1], Yin Shen[3], Rong Zhang[2], Fang Zhou[1,*] and Yong Zhang[2,*]

Author affiliations:

[1] Department of Orthopedics, Peking University Third Hospital, Beijing, P.R. China.

[2] Neuroscience Research Institute and Department of Neurobiology, School of Basic Medical Sciences, Peking University Health Science Center. Key Laboratory for Neuroscience, Ministry of Education/National Health Commission of P.R. China. IDG/McGovern Institute for Brain Research at PKU. Beijing, P.R. China.

[3] Eye Center, Renmin Hospital of Wuhan University, Wuhan, Hubei, P.R. China.

*** Corresponding author: Yong Zhang and Fang Zhou share senior authorship.**

Correspondence to: Yong Zhang, PhD

Neuroscience Research Institute, IDG/McGovern Institute for Brain Research, Peking University, Beijing, China, 100191.

E-mail: yongzhang@hsc.pku.edu.cn; https://orcid.org/0000-0001-8765-7037.

Correspondence may also be addressed to: Fang Zhou, Professor

Department of Orthopedics, Peking University Third Hospital, Beijing, China, 100191.

E-mail: zhouf@bjmu.edu.cn; https://orcid.org/0000-0002-7775-069X

Supplementary materials display items:

Fig. S1

Fig. S2

Fig. S3

Fig. S4

Fig. S5

Fig. S6

Video 1

Video 2

Video 3

Video 4

Table 1

Table 2

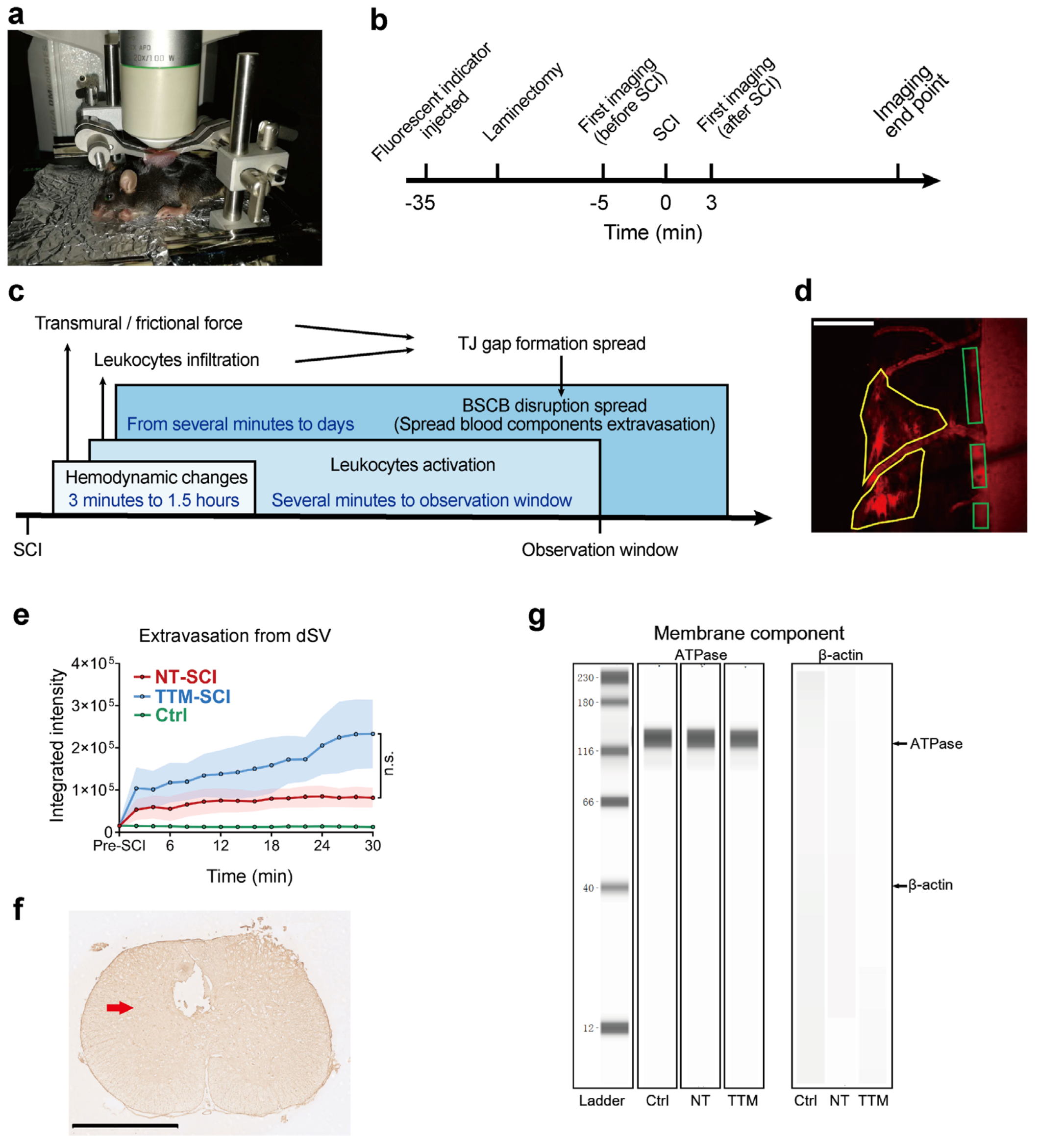

Fig. S1 **a** A homemade imaging installation. The chamber was cemented by dental cement after using the clamps to hold the vertebral column of the mouse. This installation was used for rapid imaging of the injury site before and after SCI. **b** A schematic timeline of in vivo imaging. **c** Temporal explanation of the steps of BSCB disruption after SCI. **d** Representative maximum projection about the extravasation of 40kDa TRITC-dextran 30 min post-SCI. The frames were manually drawn to quantify the extravascular fluorescence density leaked from dSV (green frame) or dAV (yellow frame) in ImageJ (NIH). **e** Quantification of fluorescence intensity extravasated from dSV in the epicenter from Fig. 1**c** (n = 10 mice; n = 6 in ctrl group). Data are the mean ± SEM, n.s. no significance; nested, general estimated equation. **f** Representative immunohisto-chemistry images of serum immunoglobulin (IgG) extravasation near the

epicenter. A cyst (red arrow) appeared 4 hours post-SCI. Scale bar = 1 mm. **g** The membrane component obtained by the Mem-PER kit (Pierce) was detected by ATPase and β-actin antibodies. ATPase is a pump of hydrogen and potassium ions located on the membrane and was used as a membrane protein loading control; the β-actin was used as a plasma protein loading control. The loading lysis was diluted to 10^{-3} μg/μl by the optimized detection concentration for ATPase.

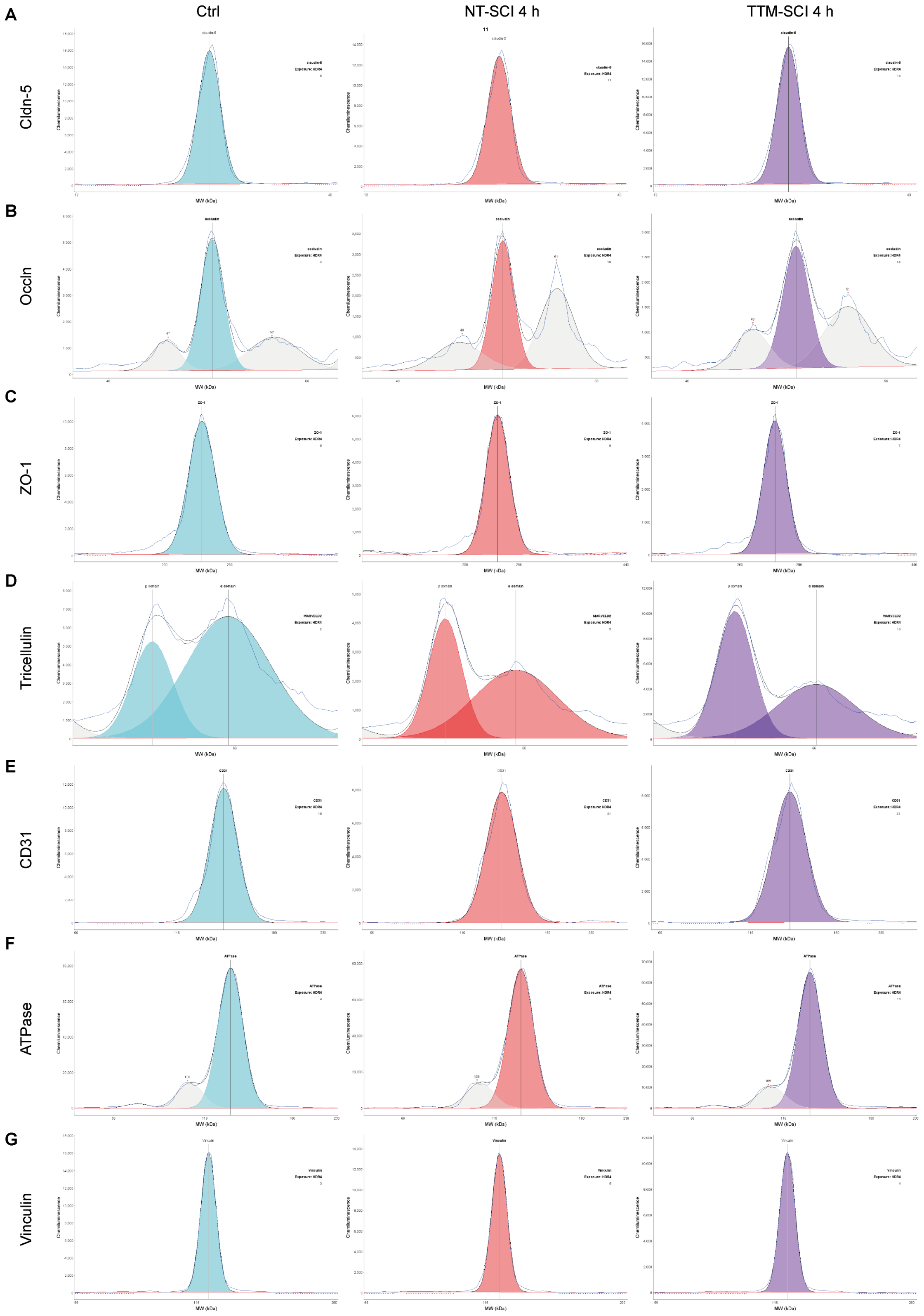

Fig. S2 Representing graph view about chemiluminescence intensity of junctional proteins measured by automated capillary western assay 4 hours post-SCI. **a** claudin-5; **b** occludin; **c** ZO-1; **d** tricellulin; **e** CD31; **f** ATPase; **g** vinculin. Vinculin was used as a

loading control for ZO-1, and ATPase was used as a loading control for the other proteins. The expression of proteins was calculated automatically by the peak-area method (colored area) in Compass software (Simple Western) (n = 5 mice)

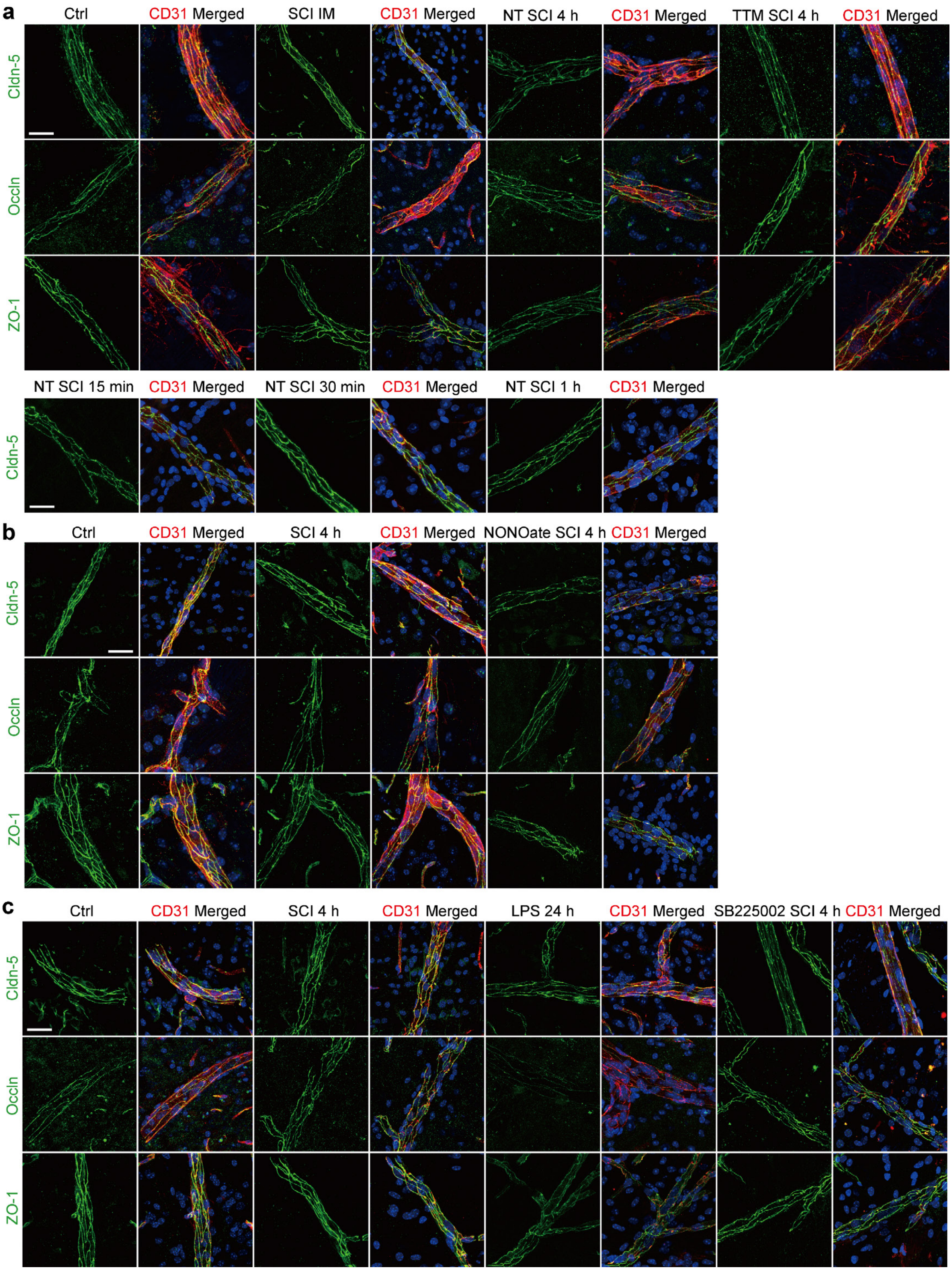

Fig. S3 Gap formation in TJs at non-capillaries small vessels post-SCI. **a**–**c** Representative images of claudin-5 (cldn-5), occludin (occln) and ZO-1 at non-capillary small vessels post-SCI. The same images were merged with CD31. Scale bar = 25 μm. The gaps in TJs were detected on these vessels. (**a**) TJs on small vessels at different time-point post-SCI with NT or TTM. (n = 5 mice, total 148, 198, 156, 166

vessels each column respectively (upper panel); cldn-5 on spinal cord 15, 30, 60 minutes post-SCI (lower panel), total 58, 63, 68 vessels respectively). (**b**) TJs at small vessels manipulated with NONOate 4 hours post-SCI. (*n* = 5 mice, total 76, 80, 136 vessels respectively). (**c**) TJs at the small vessels 24 hours after LPS injection or manipulated with CXCR2 antagonist SB225002 4 hours post-SCI. (*n* = 5 mice, total 114, 121, 118, 167 vessels respectively)

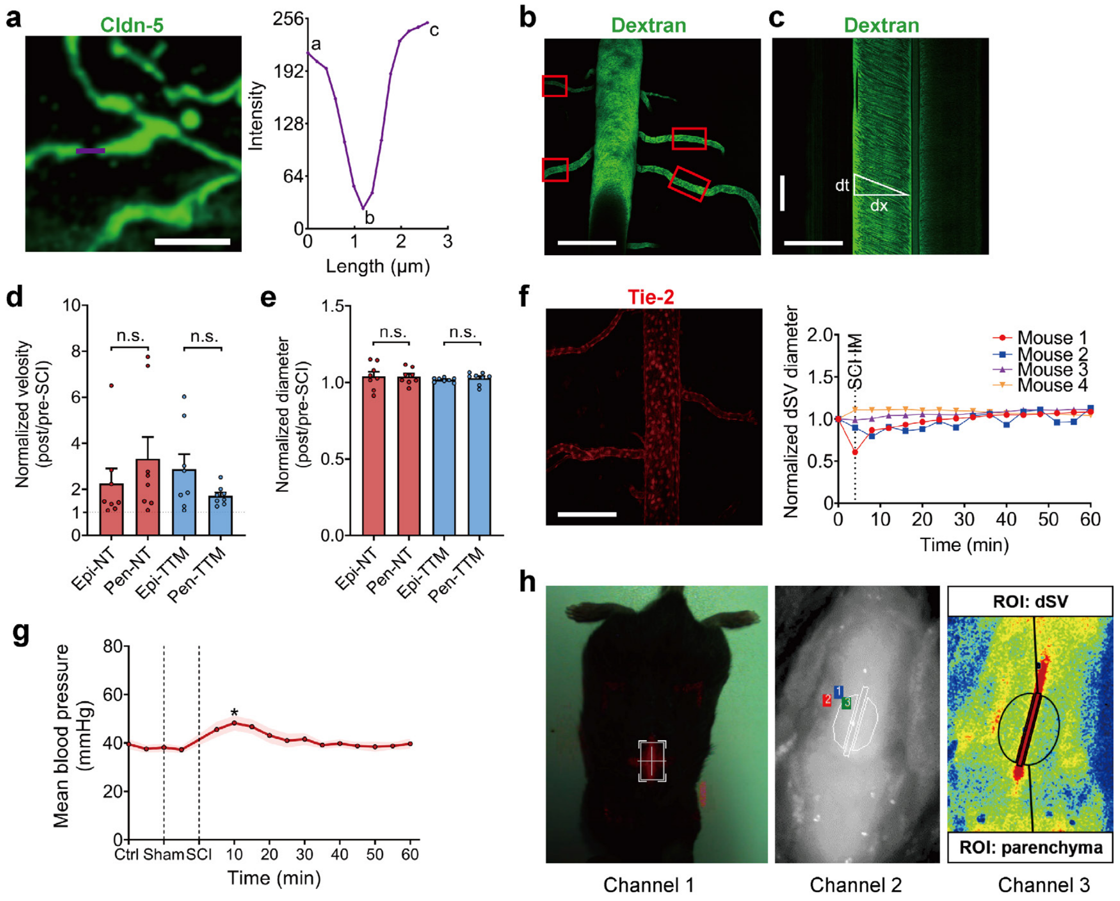

Fig. S4 a Representative images about gaps on TJs at small vessels post-SCI (left). The gap was measured by the line intensity method (purple line) in LasX software (Leica). The discontinuity characterized as a more than 70% decrease in fluorescence intensity (right image, $2b/(a+c)<30\%$) was defined as a gap. Scale bar = 5 μm. **b** Representative images about the ROIs (red frame). ROI was delineated 100 μm in width on each dAV, approximately 100-150 μm away from the connection with the dSV. The average diameter was calculated in each ROI. Scale bar = 200 μm. **c** Representative kymographs of blood flow in dAVs obtained by two-photon microscopy. Dark diagonal streaks in the figures were caused by red blood cells traveling along the vessels. The slope of the streak equals the velocity (velocity=dx⁄dt). **d** The blood flow velocity of dAVs was normalized by that measured in the same ROI pre-SCI in epicenter and penumbra from Fig. 4**b**. **e** The diameter of dAV was normalized by that measured in the same ROI pre-SCI in epicenter and penumbra from Fig. 4**e**. **f** In vivo imaging about the dSV on Tie2-tdTomato transgenic mouse (left). The diameter of dSV was observed during an hour post-SCI and normalized by self-comparison (right) (n = 5 mice, one mouse was

abandoned for thrombosis formation in dAV). **g** Tail-cuff the mean blood pressure of the mice after laminectomy (sham) and SCI (*n* = 9 mice). **h** Representative perfusion images of the spinal cord captured by non-contact laser Doppler flowmetry (Perimed). ROIs were deliberately traced with black lines. The redshift indicated more red blood cell movement. Data are the mean ± SEM; n.s. no significance, *$P < 0.05$, nested, one-way ANOVA (**d** and **e**); the Mann–Whitney test (**g**)

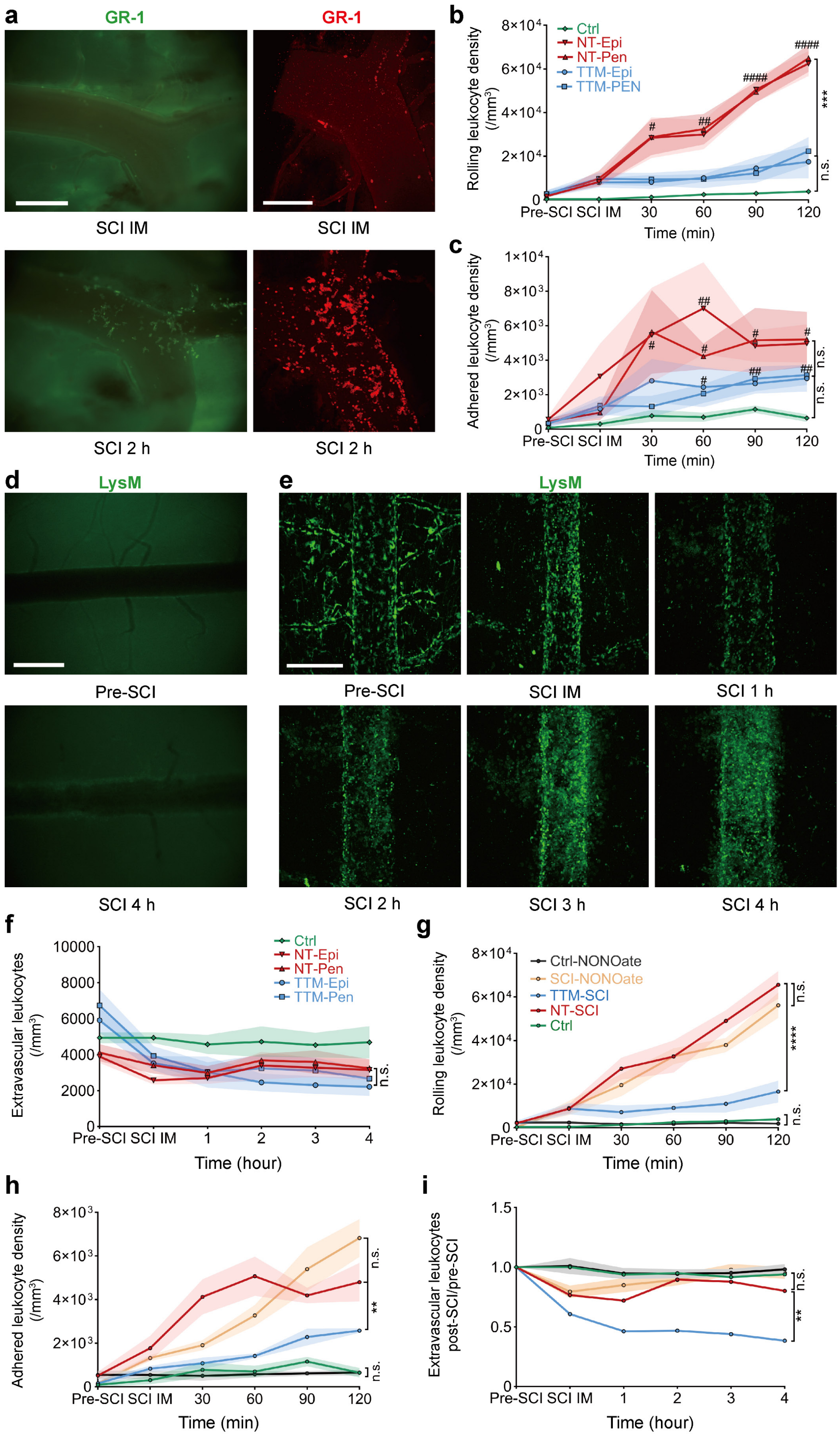

Fig. S5 a Representative images about the PE-conjugated GR-1 labeled leukocytes

were activated at the vascular junction between the dSV and spinal radial vein post-SCI under a CCD camera (left) and two-photon microscope (right). Scale bar: left = 250 μm, right = 100 μm. Many leukocytes quickly got deformed or connected to other leukocytes. (*n* = 3 mice, these data were not brought into the result in (Fig. 6**b**, **c**). **b** Density of leukocytes rolling on the lumen of dSV in epicenter and penumbra from (Fig. 6**b**). **c** Density of leukocytes adhered on the lumen of dSV in epicenter and penumbra from Fig. 6**c**. **d**, **e** Images about the most extreme case of YFP-positive cells adhered to the lumen. The ROI under CCD camera (**d**) and under two photon microscope (**e**). The extravascular LysM positive cells decreased over time, and the outline of dAVs gradually became invisible. Scale bar: left = 150, right = 200 μm. **f** YFP labeled leukocytes and/or microglia in epicenter and penumbra from Fig. 6**e** (*n* = 6 mice). **g**, **h** Density of leukocytes rolling or adhering on the lumen of dSV. **i** Density of YFP labeled leukocytes and/or microglia in the extravascular area. The vasoactive agent NONOate does not have an apparent effect on leukocyte transmigration (**g**–**i**) (*n* = 5 to 6 mice). Data are the mean ± SEM; n.s. no significance, #$P < 0.05$, ##$P < 0.01$, ####$P < 0.0001$. nested, Kruskal–Wallis test and two-way ANOVA (**b**, **c**, **f**–**i**)

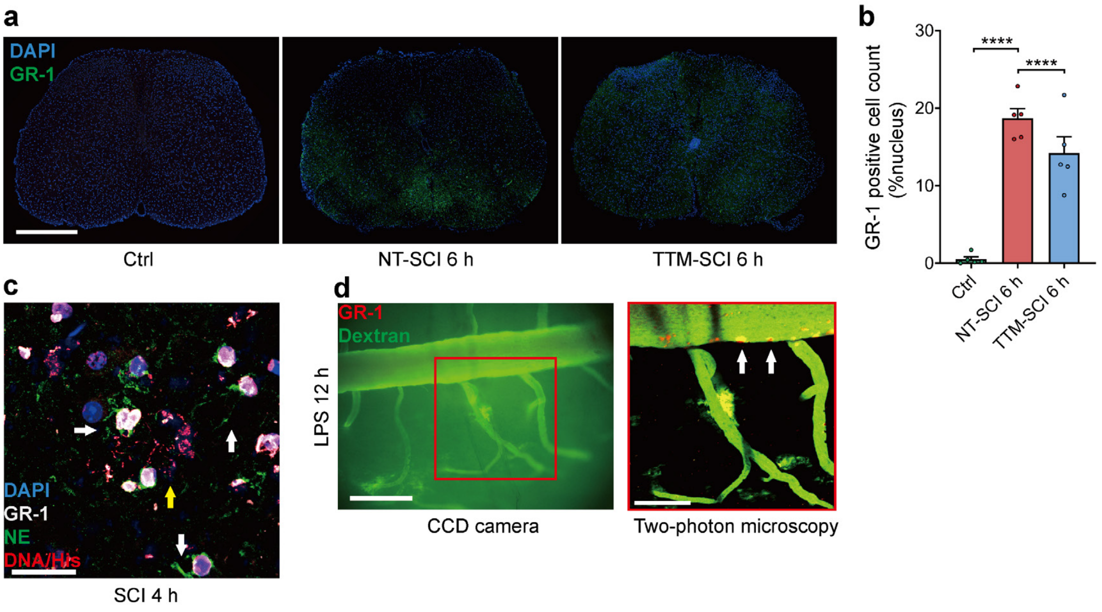

Fig. S6 a Representative images of GR-1 positive leukocytes (green) on cross-sections 6 hours post-SCI. Scale bar = 500 μm. **b** Quantification of GR-1 positive leukocytes in multiple spinal segments 6 hours post-SCI. The value was adjusted by the number of DAPI positive nuclei (n = 5 mice). **c** A representative image about GR-1 labeled neutrophils (gray) released nuclear DNA/histone complex (red) and cytoplasmic neutrophils elastase (NE, green) into nearby tissue 4 hours post-SCI. The extracellular neutrophils elastase formed a spiky-like structure (white arrow), and the cell-free DNA/histone formed a cloudy-like structure (yellow arrow) (n = 7 independent experiments). Scale bar = 25 μm. **d** Representative images about AlexaFluor 700 conjugated GR-1 labeled leukocytes (red) activated after LPS injection. The plasma was labeled by 40 kDa FITC-dextran (green). The leakage related to leukocytes transmigration was less at tubular dSV (arrow) than at vascular junction between the dSV and spinal radial vein in (Fig. 8**c**) (n = 6 mice). Scale bar: left = 250 μm, right = 100 μm. Data are the mean ± SEM; *P < 0.05, *P < 0.0001. nested, one-way ANOVA (**b**)

Video 1 Blood flow stasis or blood clots appears at dAVs in the epicenter immediately post-SCI. Time-lapse fluorescence microscopy was captured under a CCD camera by intravenous injection of 150 kDa FTIC-dextran. Some blood clots could form to hinder the blood flow. This phenomenon usually lasts less than 5 minutes and could be repeatedly observed in independent experiments

Video 2 Blood flow stasis is eliminated by blood flow acceleration in dAVs shortly post-SCI. The ROI in this video was the same region as shown in Additional file 2: Video 1 several minutes later. The stasis or blood clot was quickly eliminated, and vessel recirculation occurred along with blood flow acceleration. The pathological blood acceleration arose, and the blood flow stasis or blood clots were eliminated. The blood components started to leak in the same time frame

Video 3 Blood flow acceleration post-SCI was partly slowed down by topical application of NONOate. Time-lapse fluorescence microscopy was captured by CCD camera by intravenous injection of 150 kDa FTIC-dextran

Video 4 Activated leukocytes roll and adhere on the vessel endothelium. Time-lapse fluorescence microscopy showed by intravenous injection of PE-conjugated GR-1 (Ly6G/C) to label leukocytes and 150 kDa FTIC-dextran to label plasma. Some leukocytes have already transferred through the BSCB into parenchyma or attached to the dura

Table 1 Antibodies or fluorescence materials used in this study

Name	Manufacturer	Stock Nos.	Concentration	Dosage	Application
CD31	Abcam	ab119341	1:200	NA	IF
CD31	Abcam	ab222783	1:200	NA	WB
Claudin-5	Bioworld	BS1069	1:100/1:100	NA	IF/WB
Claudin 5	Abcam	ab131259	1:100	NA	WB
ZO-1	Invitrogen	40-2200	1:100/1:200	NA	IF/WB
Occludin	Invitrogen	40-4700	1:150/1:100	NA	IF/WB
Occludin	Abcam	ab167161	1:100	NA	WB
MARVELD2	Invitrogen	48-8400	1:100	NA	WB
IgG-Fc	Bethyl	A90-131A	1:800	NA	IHC
Vinculin	Cell signaling	13901	1:400	NA	WB
Vinculin	Abcam	ab129002	1:400	NA	WB
ATPase	Abcam	ab76020	1:500	NA	WB
β-actin	Cell signaling	4970	1:500	NA	WB
GR1	BD bioscience	553122	1:50	NA	IF
CD11b	Abcam	ab184308	1:300	NA	IF
DNA/Histon	Millipore	MAB3864	1:300	NA	IF
Neutrophil Elastase	Invitrogen	PA5-87158	1:50	NA	IF
Histone H3	Abcam	ab5103	1:150	NA	IF
NeuN	Millipore	MAB377	1:200	NA	IF
Ly6G	BD bioscience	553122	Not found	NA	IF
Goat anti-Rabbit Alexa488	Abcam	ab150081	1:500	NA	IF
Goat anti-Hamster Alexa568	Abcam	ab175716	1:500	NA	IF

Goat anti-mouse Alexa594	Abcam	ab150120	1:1000	NA	IF
Goat anti-Rabbit Alexa647	Abcam	ab150083	1:1000	NA	IF
Goat anti-Rat Alexa488	Abcam	ab150157	1:1000	NA	IF
Goat anti-Rat Alexa647	Abcam	ab150159	1:500	NA	IF
Rhodamine 6G	Sigma	83697	0.1% (w/v)	1 mg/kg	In Vivo
FITC-WGA	Sigma	L4895	1 mg/ml	6 mg/kg	In Vivo
FITC-dextran 150 kDa	Sigma	69658-1G	5% (w/v)	20 mg/g	In Vivo
TRITC-dextran 155 kDa	Sigma	T1287-100MG	5% (w/v)	20 mg/g	In Vivo
FITC-dextran 40 kDa	Sigma	FD40-250MG	5% (w/v)	20 mg/g	In Vivo
TRITC-dextran 40 kDa	Sigma	42874-1G	5% (w/v)	20 mg/g	In Vivo
PE-Ly6G	BD bioscience	561104	50 µg/ml	200 µg/kg	In Vivo
FITC-GR1	Biolegend	108405	0.2 mg/ml	250 µg/kg	In Vivo
PE-GR1	Biolegend	108407	0.2 mg/ml	200 µg/kg	In Vivo
Alexa700-GR1	Biolegend	108421	0.2 mg/ml	200 µg/kg	In Vivo
Alexa647-GR1	Biolegend	108420	0.2 mg/ml	200 µg/kg	In Vivo
FITC-CD31	BD bioscience	561813	Not found	Not found	In Vivo

Table 2 Statistical test results in this study
