# Supplementary material for: Pathological hemodynamic changes and leukocyte transmigration disrupt the blood–spinal cord barrier after spinal cord injury
Source: J Neuroinflammation. 2023 May 20;20:118. doi: 10.1186/s12974-023-02787-w (PMC10200062; doi:10.1186/s12974-023-02787-w)

Pathological hemodynamic changes and leukocyte transmigration disrupt the blood–spinal cord barrier after spinal cord injury

Rubing Zhou[1,2], Junzhao Li[2], Zhengyang Chen[1], Ruideng Wang[1], Yin Shen[3], Rong Zhang[2], Fang Zhou[1,*] and Yong Zhang[2,*]

Author affiliations:

[1] Department of Orthopedics, Peking University Third Hospital, Beijing, P.R. China.

[2] Neuroscience Research Institute and Department of Neurobiology, School of Basic Medical Sciences, Peking University Health Science Center. Key Laboratory for Neuroscience, Ministry of Education/National Health Commission of P.R. China. IDG/McGovern Institute for Brain Research at PKU. Beijing, P.R. China.

[3] Eye Center, Renmin Hospital of Wuhan University, Wuhan, Hubei, P.R. China.

*** Corresponding author: Yong Zhang and Fang Zhou share senior authorship.**

Correspondence to: Yong Zhang, PhD

Neuroscience Research Institute, IDG/McGovern Institute for Brain Research, Peking University, Beijing, China, 100191.

E-mail: yongzhang@hsc.pku.edu.cn; https://orcid.org/0000-0001-8765-7037.

Correspondence may also be addressed to: Fang Zhou, Professor

Department of Orthopedics, Peking University Third Hospital, Beijing, China, 100191.

E-mail: zhouf@bjmu.edu.cn; https://orcid.org/0000-0002-7775-069X

Additional remarks

The proteins levels were measured with an automatic capillary western blot system (Simple Western, ProteinSimple). These virtual images of the bands were used as representative images, the peak area of chemiluminescence intensity (shown below) automatically computed by Compass software (ProteinSimple) was used to quantify the result of the capillary western blot. Since the abundance of reference proteins was much higher than the target proteins, the detection of reference proteins and target proteins was performed in separate plate wells according to the manufacturer's advice.

Claudin-5

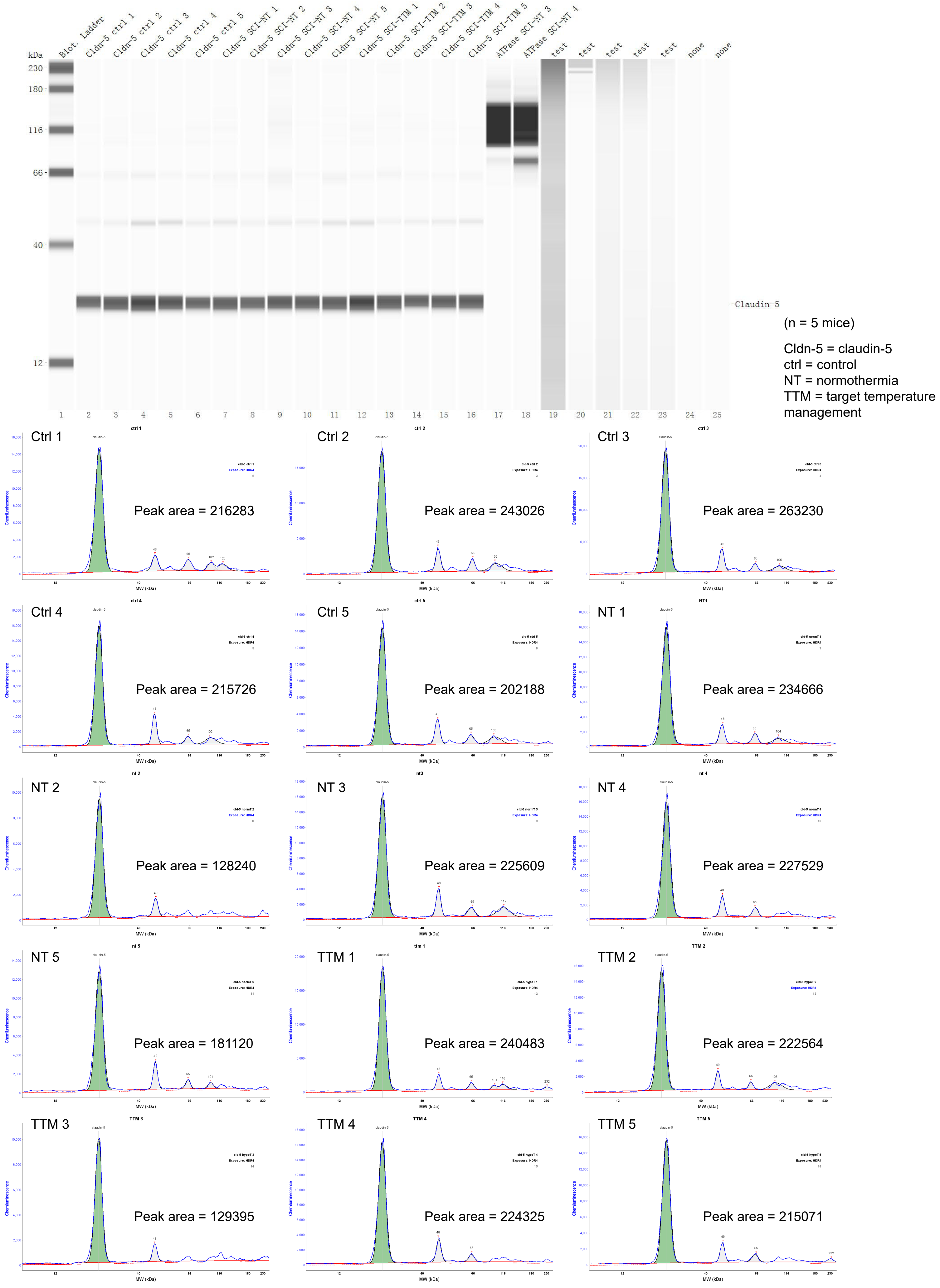

kDa
230
180
116
66
40
12
Biot. Ladder
Cldn-5 ctrl 1
Cldn-5 ctrl 2
Cldn-5 ctrl 3
Cldn-5 ctrl 4
Cldn-5 ctrl 5
Cldn-5 SCI-NT 1
Cldn-5 SCI-NT 2
Cldn-5 SCI-NT 3
Cldn-5 SCI-NT 4
Cldn-5 SCI-NT 5
Cldn-5 SCI-TTM 1
Cldn-5 SCI-TTM 2
Cldn-5 SCI-TTM 3
Cldn-5 SCI-TTM 4
Cldn-5 SCI-TTM 5
ATPase SCI-NT 3
ATPase SCI-NT 4
test
test
test
test
test
none
none
-Claudin-5
1 2 3 4 5 6 7 8 9 10 11 12 13 14 15 16 17 18 19 20 21 22 23 24 25
(n = 5 mice)
Cldn-5 = claudin-5
ctrl = control
NT = normothermia
TTM = target temperature management
Ctrl 1
Peak area = 216283
Ctrl 2
Peak area = 243026
Ctrl 3
Peak area = 263230
Ctrl 4
Peak area = 215726
Ctrl 5
Peak area = 202188
NT 1
Peak area = 234666
NT 2
Peak area = 128240
NT 3
Peak area = 225609
NT 4
Peak area = 227529
NT 5
Peak area = 181120
TTM 1
Peak area = 240483
TTM 2
Peak area = 222564
TTM 3
Peak area = 129395
TTM 4
Peak area = 224325
TTM 5
Peak area = 215071
MW (kDa)
Chemiluminescence

Occludin

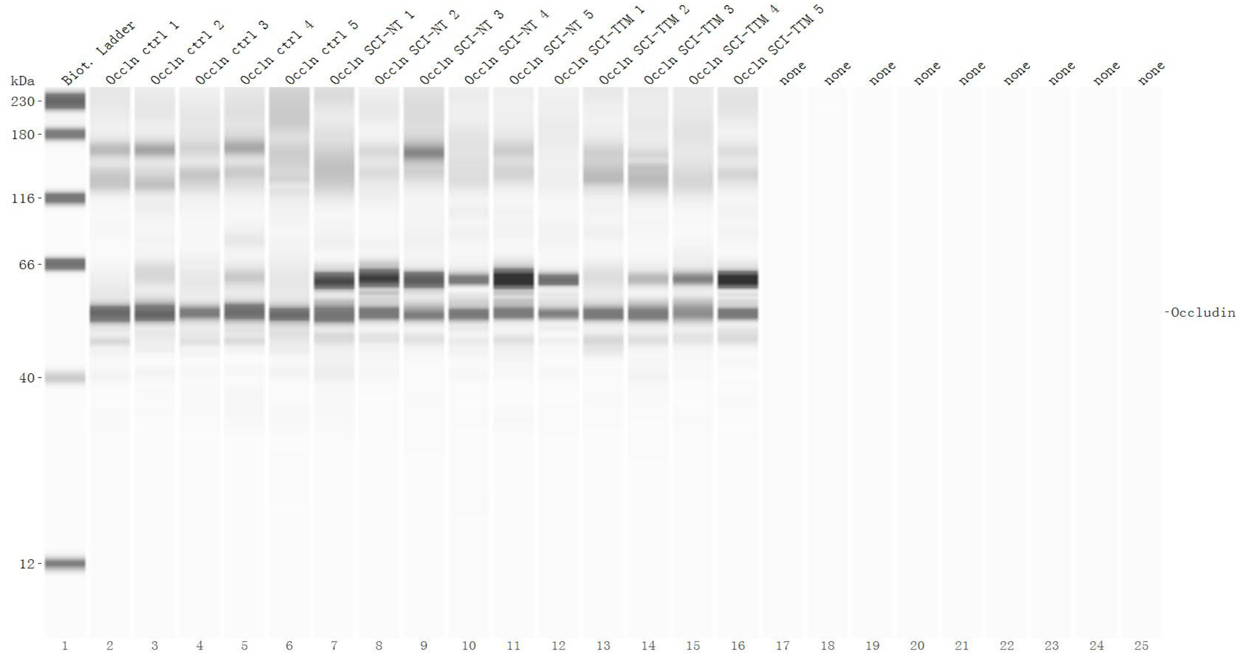

(n = 5 mice)
Occln = occludin

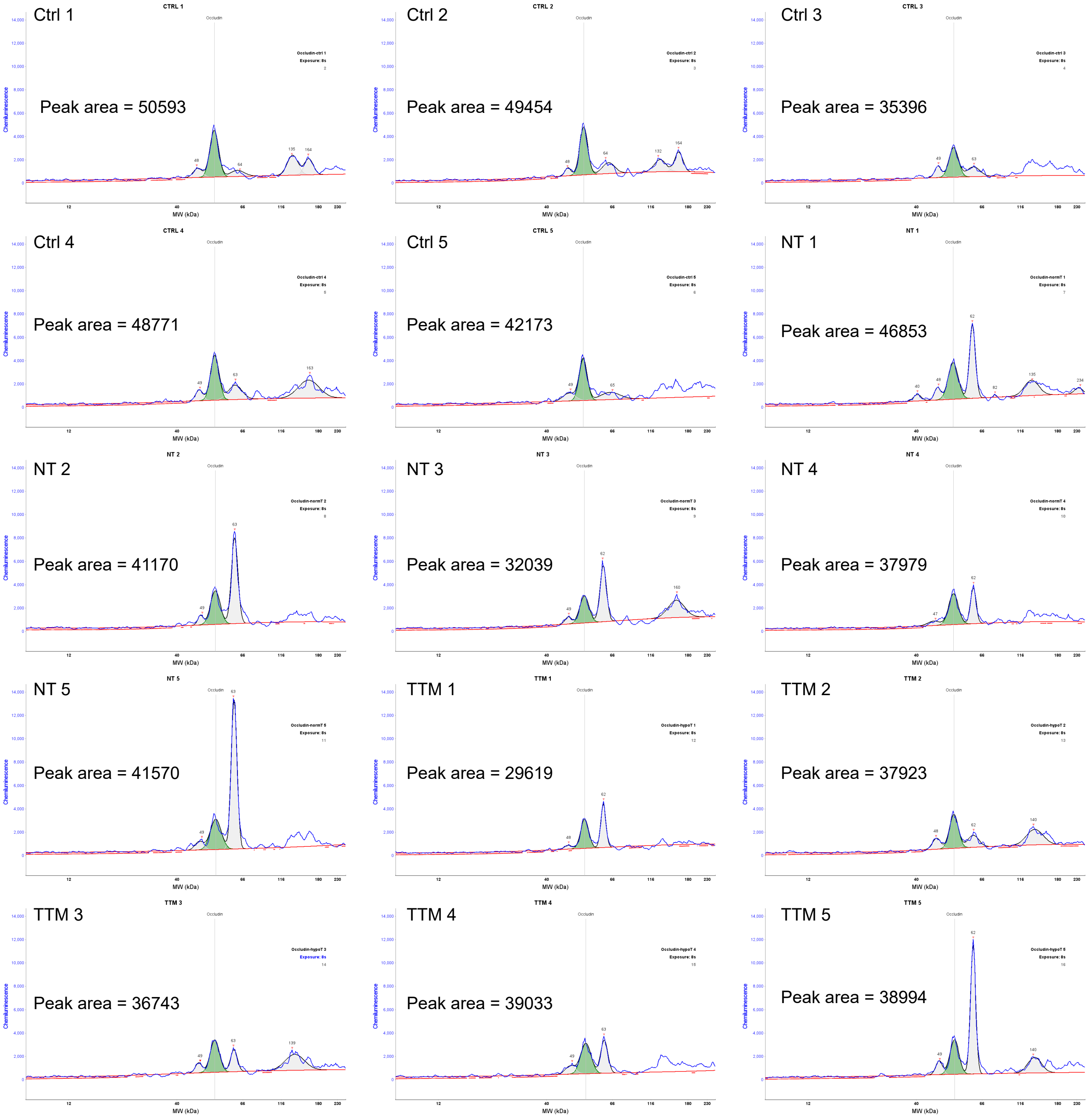

ZO-1

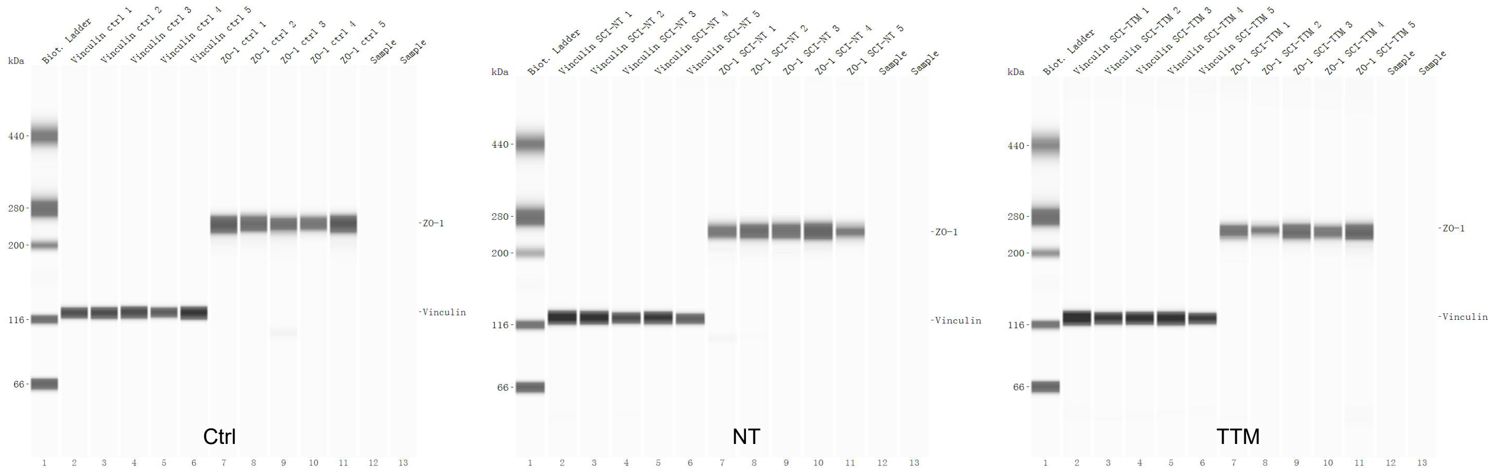

Ctrl
NT
TTM
ZO-1
Vinculin

(n = 5 mice)

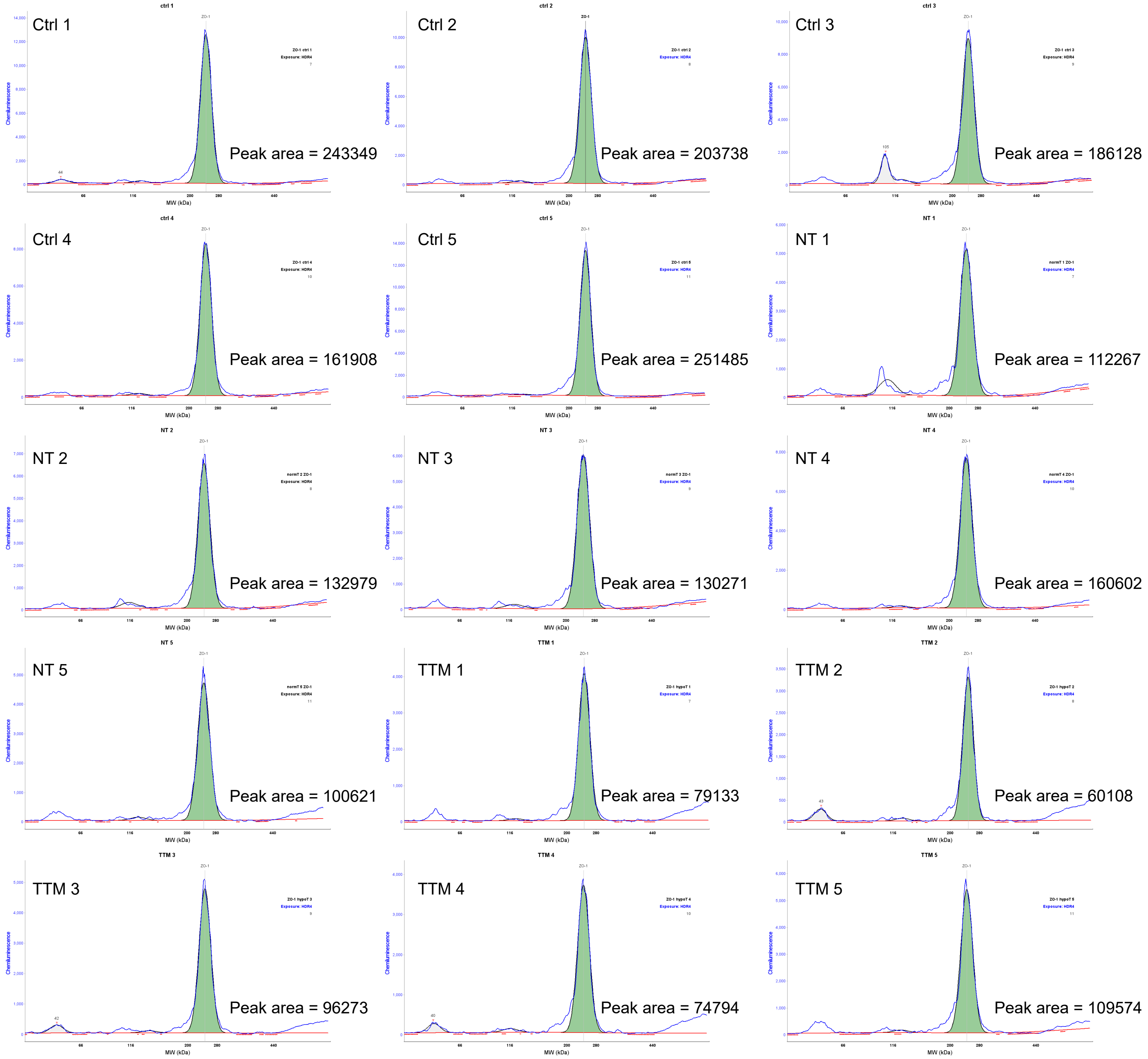

Ctrl 1
Peak area = 243349
Ctrl 2
Peak area = 203738
Ctrl 3
Peak area = 186128
Ctrl 4
Peak area = 161908
Ctrl 5
Peak area = 251485
NT 1
Peak area = 112267
NT 2
Peak area = 132979
NT 3
Peak area = 130271
NT 4
Peak area = 160602
NT 5
Peak area = 100621
TTM 1
Peak area = 79133
TTM 2
Peak area = 60108
TTM 3
Peak area = 96273
TTM 4
Peak area = 74794
TTM 5
Peak area = 109574

Tricellulin

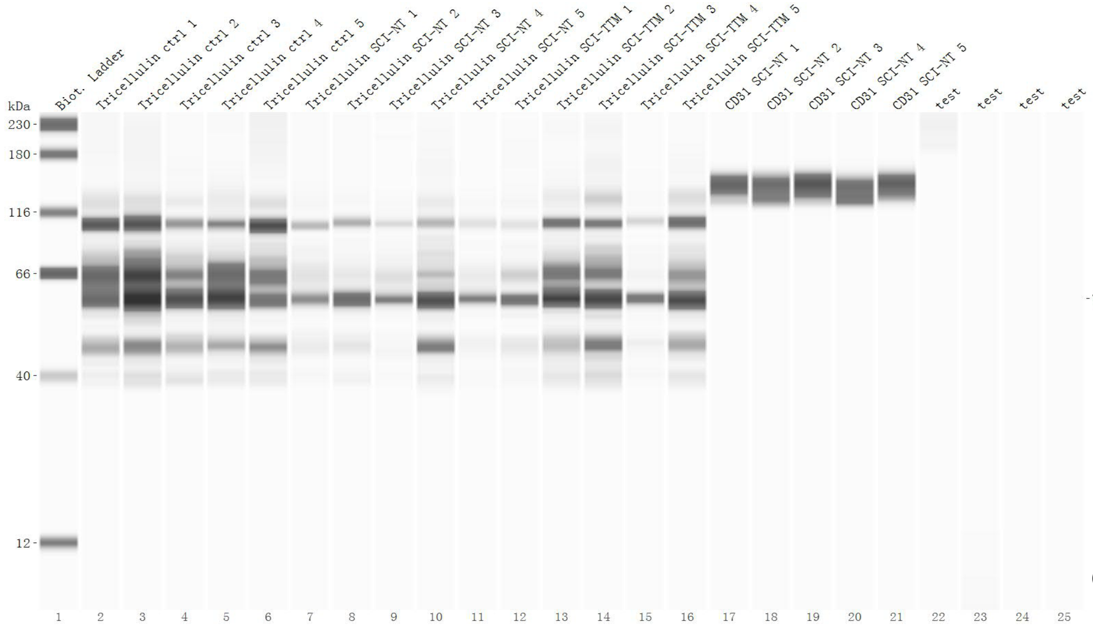

(n = 5 mice)

(Tricellulin specifically has two bands)

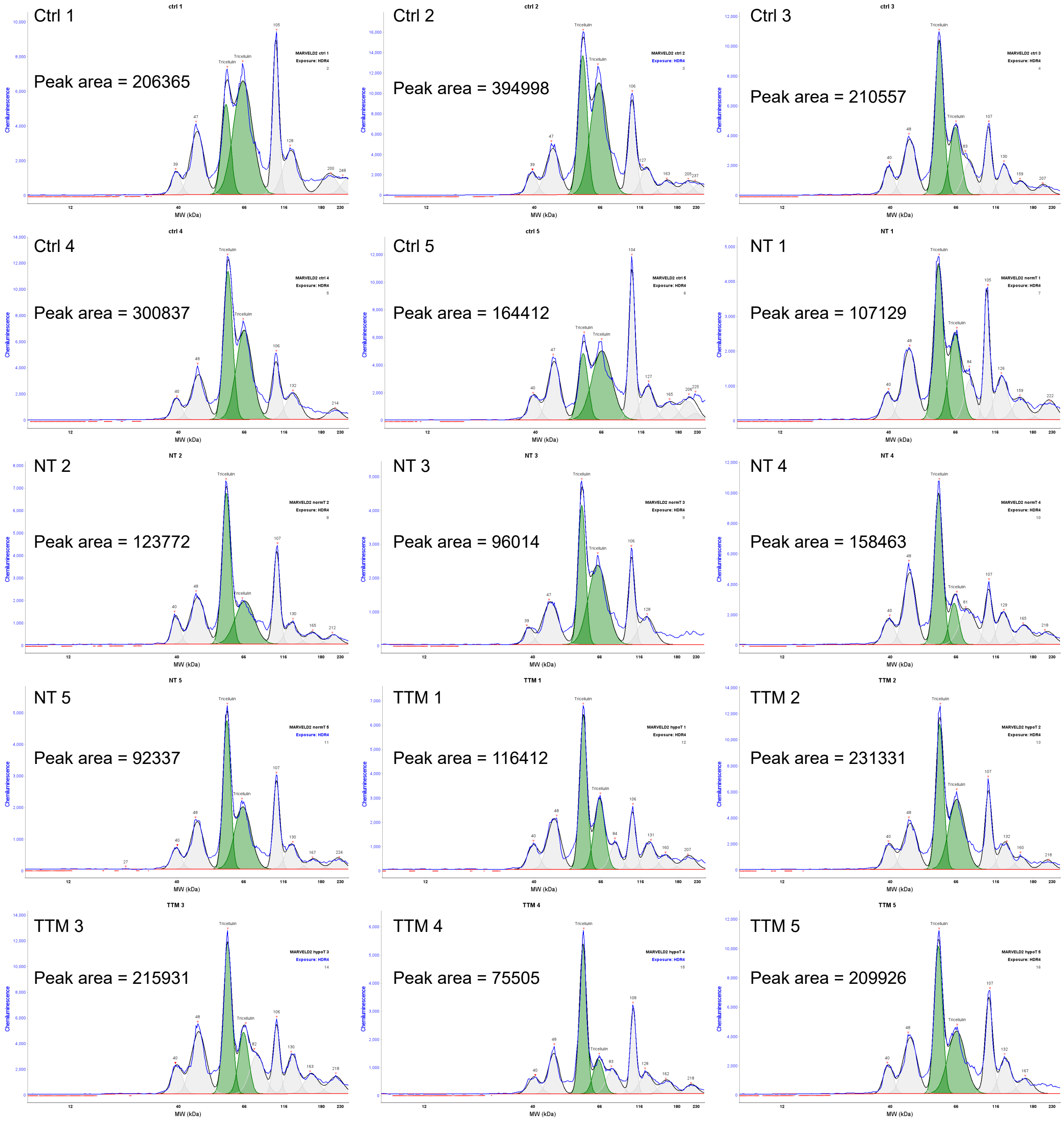

ATPase

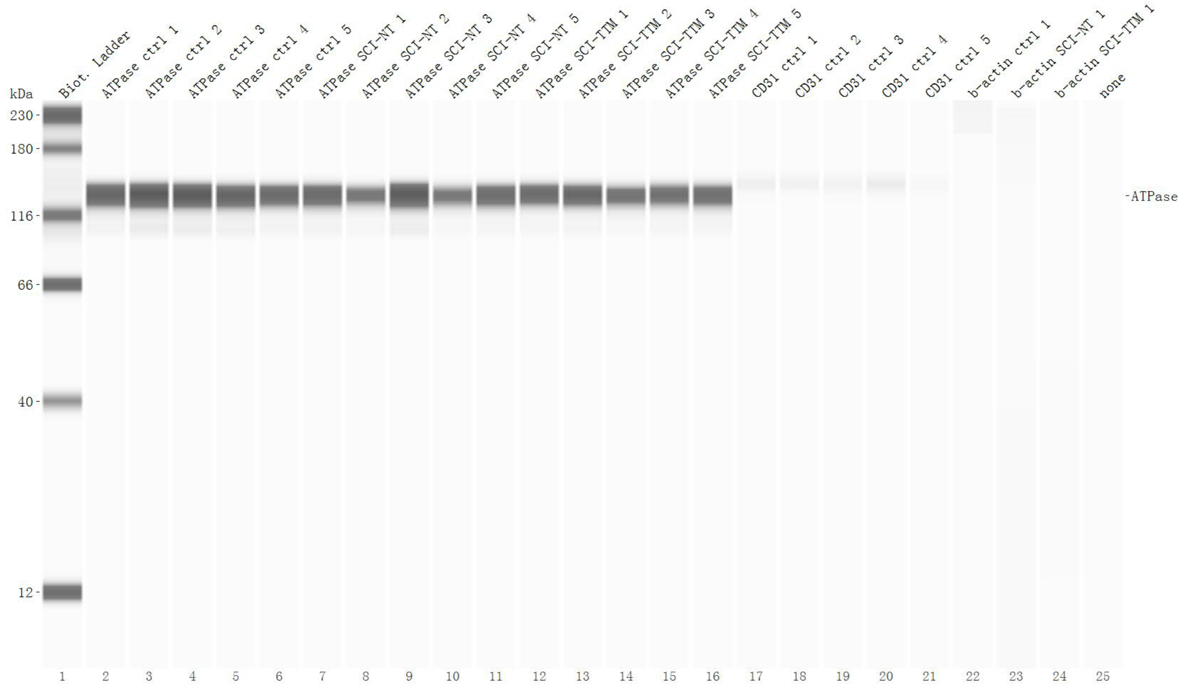

(n = 5 mice)

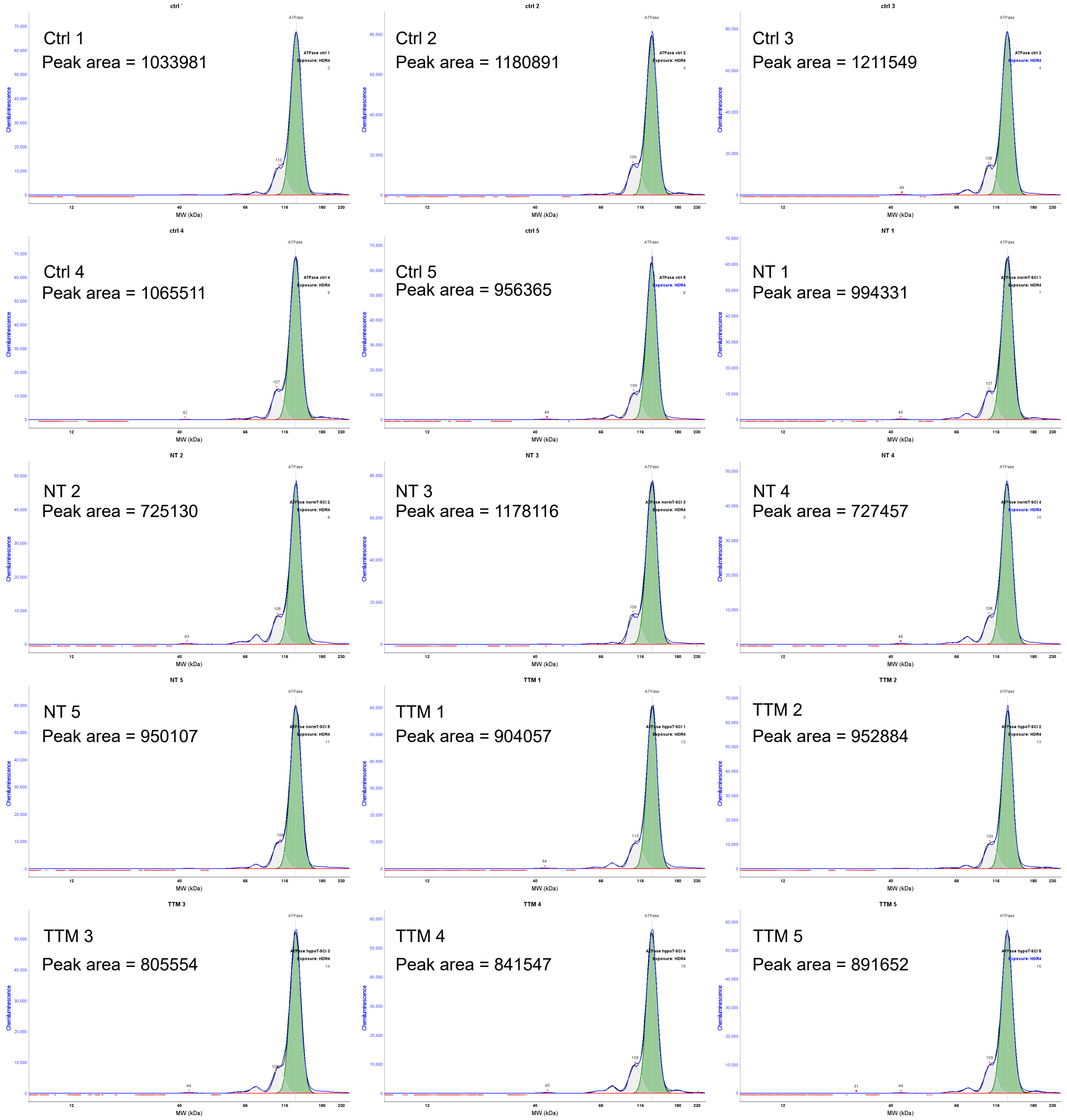

Vinculin

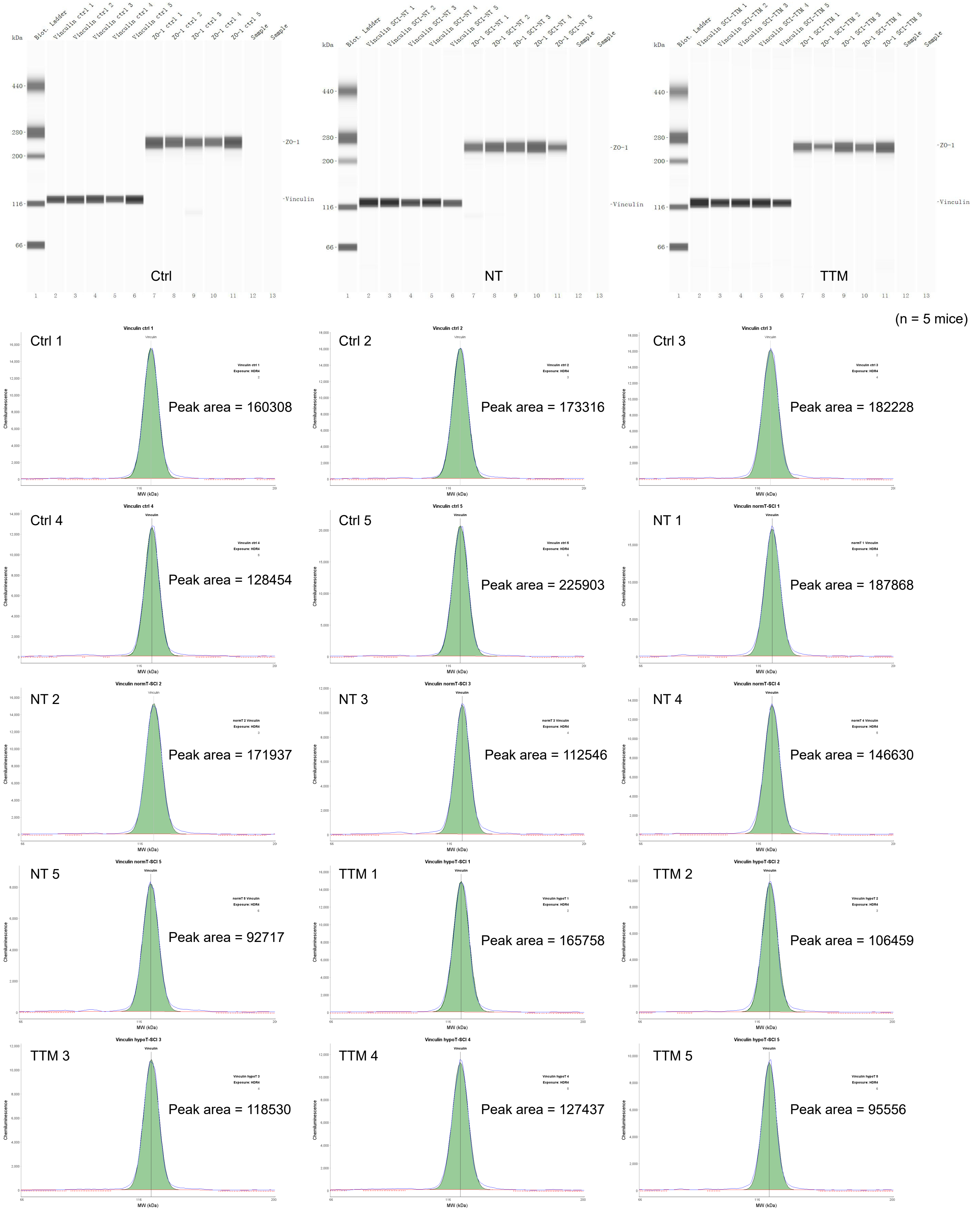

Ctrl
NT
TTM
ZO-1
Vinculin
(n = 5 mice)
Ctrl 1
Peak area = 160308
Ctrl 2
Peak area = 173316
Ctrl 3
Peak area = 182228
Ctrl 4
Peak area = 128454
Ctrl 5
Peak area = 225903
NT 1
Peak area = 187868
NT 2
Peak area = 171937
NT 3
Peak area = 112546
NT 4
Peak area = 146630
NT 5
Peak area = 92717
TTM 1
Peak area = 165758
TTM 2
Peak area = 106459
TTM 3
Peak area = 118530
TTM 4
Peak area = 127437
TTM 5
Peak area = 95556

CD31

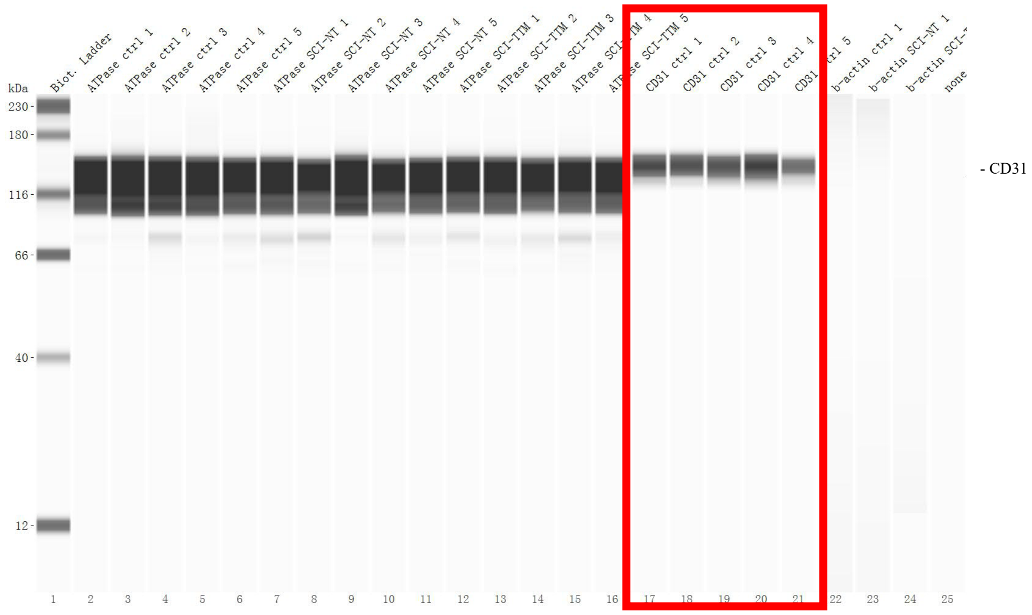

Ctrl

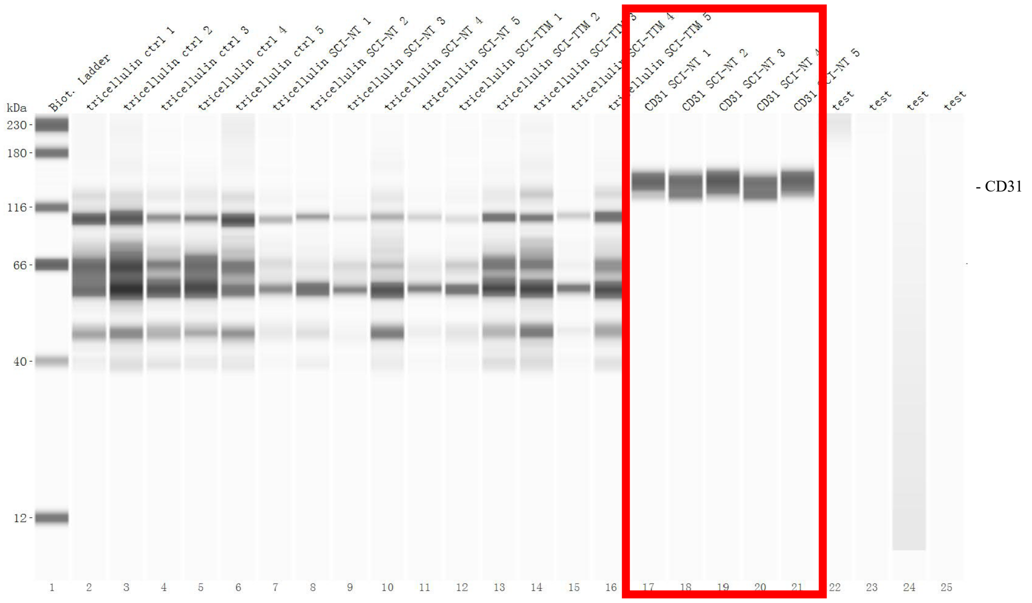

NT

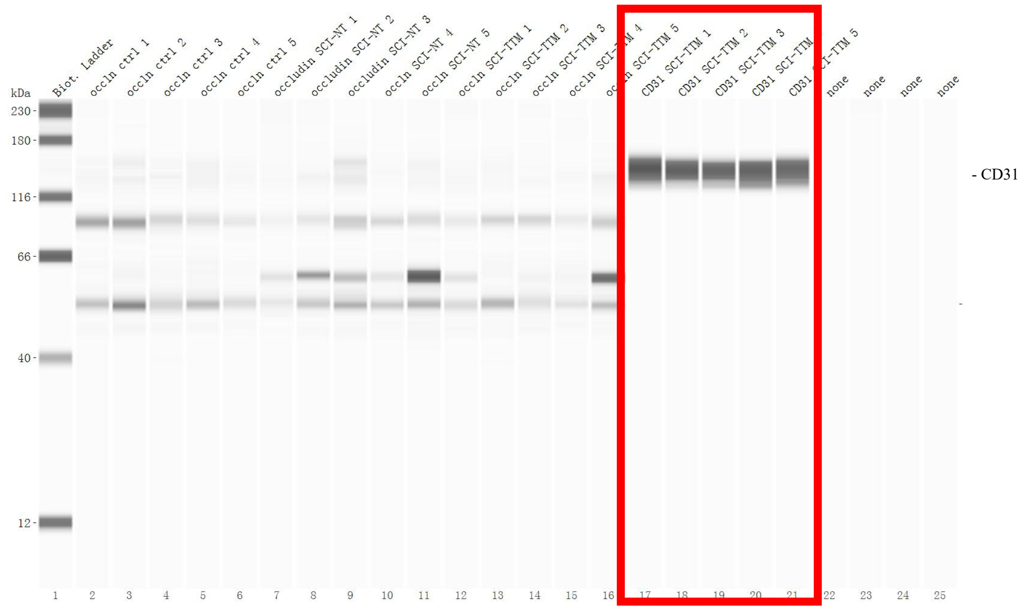

TTM

(n = 5 mice)

CD31

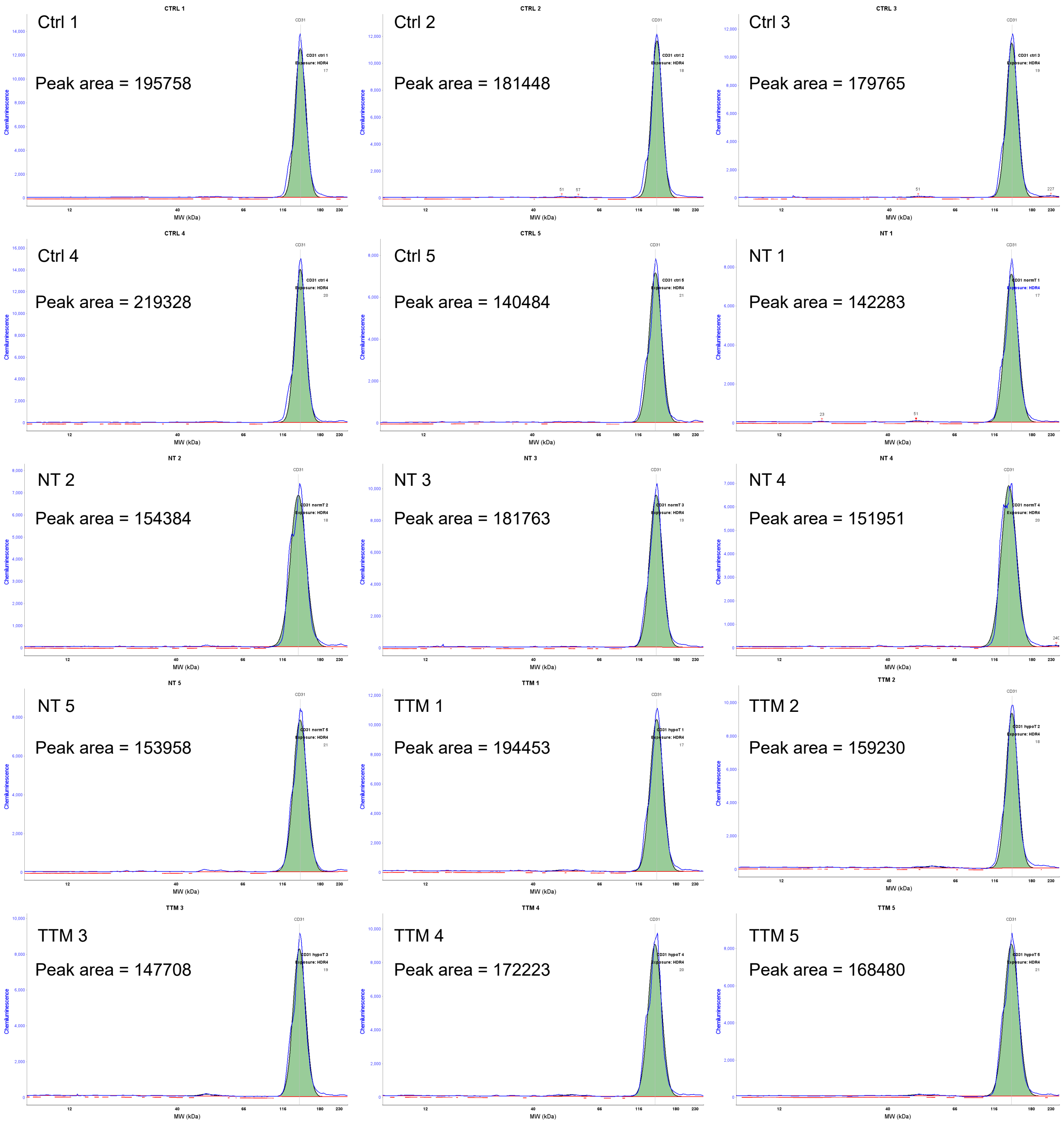
Ctrl 1
Peak area = 195758
Ctrl 2
Peak area = 181448
Ctrl 3
Peak area = 179765
Ctrl 4
Peak area = 219328
Ctrl 5
Peak area = 140484
NT 1
Peak area = 142283
NT 2
Peak area = 154384
NT 3
Peak area = 181763
NT 4
Peak area = 151951
NT 5
Peak area = 153958
TTM 1
Peak area = 194453
TTM 2
Peak area = 159230
TTM 3
Peak area = 147708
TTM 4
Peak area = 172223
TTM 5
Peak area = 168480

Claudin-5 detected by additional antibody

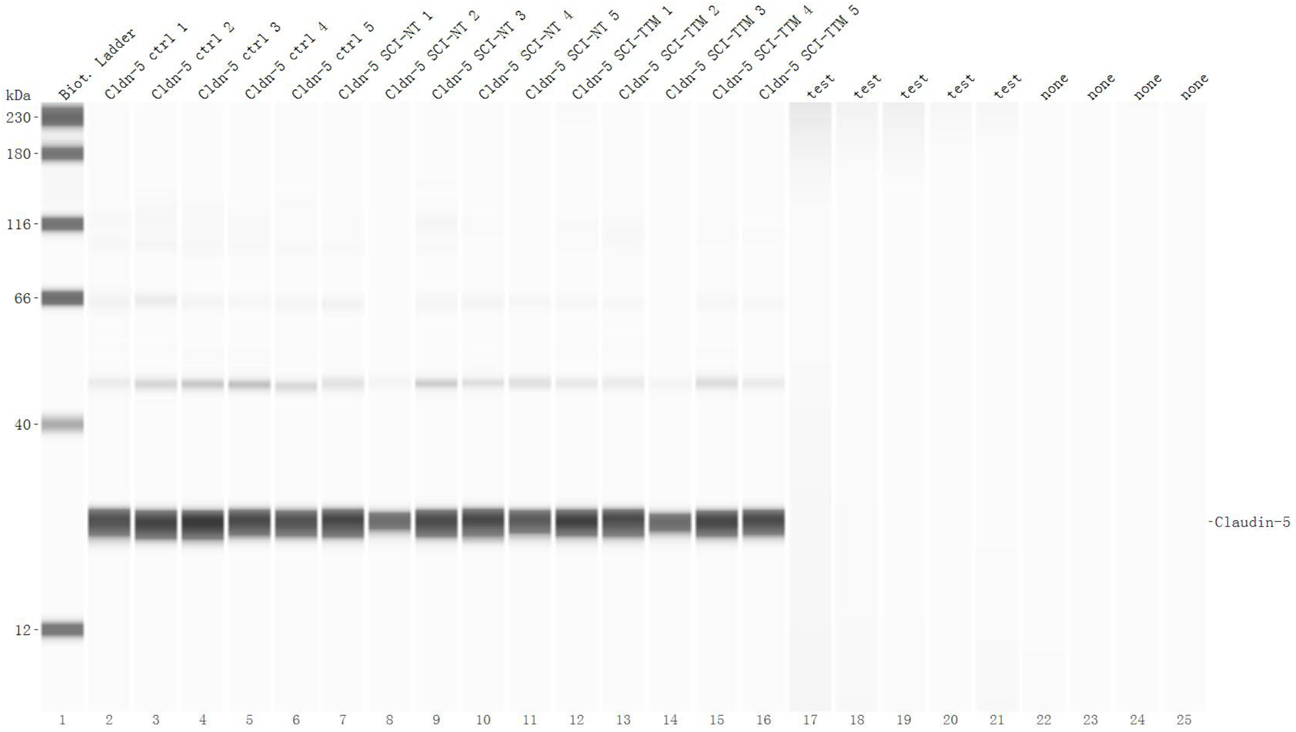

Occludin detected by additional antibody

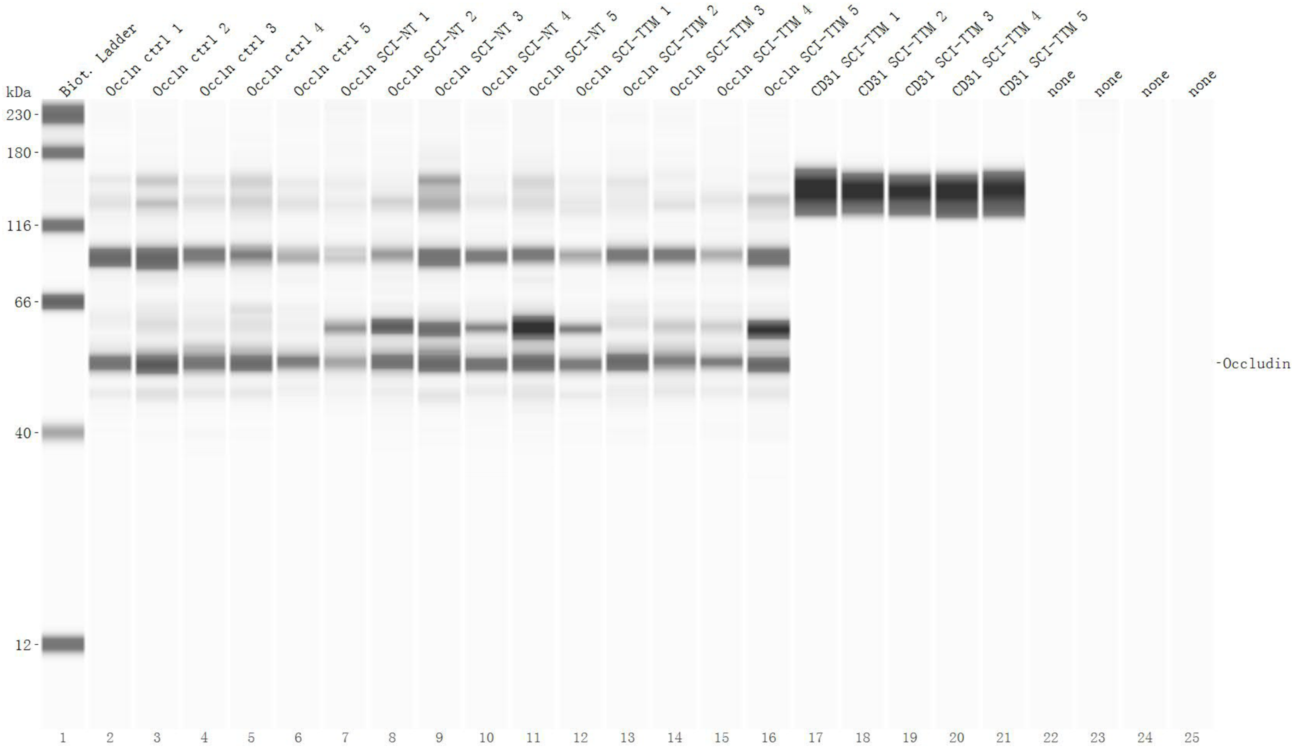

Supplement: Supplementary file 7 — Additional file 7. Full images of western blots presented in main figures. [file 12974_2023_2787_MOESM7_ESM.pdf]
